# Structure‐guided engineering of key amino acids in UGT85B1 controlling substrate and stereo‐specificity in aromatic cyanogenic glucoside biosynthesis

**DOI:** 10.1111/tpj.15904

**Published:** 2022-08-03

**Authors:** Rita Del Giudice, Natalia Putkaradze, Bruna Marques dos Santos, Cecilie Cetti Hansen, Christoph Crocoll, Mohammed Saddik Motawia, Folmer Fredslund, Tomas Laursen, Ditte Hededam Welner

**Affiliations:** ^1^ Plant Biochemistry, Department of Plant and Environmental Sciences University of Copenhagen Thorvaldsensvej 40 DK‐1871 Copenhagen Denmark; ^2^ The Novo Nordisk Foundation Center for Biosustainability Technical University of Denmark Kemitorvet 220 DK‐2800 Kgs. Lyngby Denmark; ^3^ DynaMo Center, Molecular Plant Biology, Department of Plant and Environmental Sciences University of Copenhagen Thorvaldsensvej 40 DK‐1871 Copenhagen Denmark

**Keywords:** crystal structure, dhurrin, glycosyltransferase, prunasin, rational engineering, sambunigrin, taxiphyllin

## Abstract

Cyanogenic glucosides are important defense molecules in plants with useful biological activities in animals. Their last biosynthetic step consists of a glycosylation reaction that confers stability and increases structural diversity and is catalyzed by the UDP‐dependent glycosyltransferases (UGTs) of glycosyltransferase family 1. These versatile enzymes have large and varied substrate scopes, and the structure–function relationships controlling scope and specificity remain poorly understood. Here, we report substrate‐bound crystal structures and rational engineering of substrate and stereo‐specificities of UGT85B1 from *Sorghum bicolor* involved in biosynthesis of the cyanogenic glucoside dhurrin. Substrate specificity was shifted from the natural substrate (*S*)‐*p*‐hydroxymandelonitrile to (*S*)‐mandelonitrile by combining a mutation to abolish hydrogen bonding to the *p*‐hydroxyl group with a mutation to provide steric hindrance at the *p*‐hydroxyl group binding site (V132A/Q225W). Further, stereo‐specificity was shifted from (*S*) to (*R*) by substituting four rationally chosen residues within 6 Å of the nitrile group (M312T/A313T/H408F/G409A). These activities were compared to two other UGTs involved in the biosynthesis of aromatic cyanogenic glucosides in *Prunus dulcis* (almond) and *Eucalyptus cladocalyx*. Together, these studies enabled us to pinpoint factors that drive substrate and stereo‐specificities in the cyanogenic glucoside biosynthetic UGTs. The structure‐guided engineering of the functional properties of UGT85B1 enhances our understanding of the evolution of UGTs involved in the biosynthesis of cyanogenic glucosides and will enable future engineering efforts towards new biotechnological applications.

## Significance Statement

Glycosylation of plant natural products is an important modification that enhances their stability and solubility and prevents autotoxicity. Understanding the structure–function relationship of glycosyltransferases is critical for our understanding of plant natural product biosynthesis. We provide structural insights into the features controlling substrate and stereo‐specificity of UGT85B1, the glycosyltransferase catalyzing the production of the cyanogenic glucoside dhurrin in *Sorghum bicolor*. This study will enable engineering of UGT85 enzymes for tailored glycosylation of a palette of natural products.

## INTRODUCTION

Glycosylation of natural products, catalyzed by UDP‐dependent glycosyltransferases (UGTs), is a key step in biosynthesis of all the major classes of compounds including terpenoids, alkaloids, flavonoids, glucosinolates, and cyanogenic glucosides (Thorson & Vogt, 2005). UGTs are a superfamily of enzymes that use UDP‐activated sugar donors to transfer a sugar moiety to an acceptor molecule. Glycosylation increases the solubility, improves stability, and provides diversity in terms of structure and function of the compounds (Pičmanová & Møller, 2016; Thorson & Vogt, 2005). This makes UGTs important enzymes in plant metabolism where glycosylation affects, for example, chemical defense storage, hormonal homeostasis, metabolite transportation, fruit flavor, and flower color (De Bruyn et al., 2015; Le Roy et al., 2016). These roles accordingly make UGTs of great biotechnological interest for engineering of plant traits but may also be utilized for other biotechnological applications, for example to increase solubility and diversification of pharmaceutical compounds (De Bruyn et al., 2015; Wen et al., 2021).

A large number of plants, including many crop plants, produce cyanogenic glucosides as chemical defense against herbivores through the release of toxic hydrogen cyanide (Jones, 1998; Møller, 2010). This occurs upon tissue disruption whereby otherwise spatially separated beta‐glucosidases cleave off the stabilizing sugar moiety, leading to dissociation of the cyanohydrin and release of hydrogen cyanide (Gleadow & Møller, 2014). In the cereal crop *Sorghum bicolor* (great millet) the major cyanogenic glucoside is dhurrin ((*S*)‐*p*‐hydroxymandelonitrile glucoside), which may constitute up to 30% of the dry weight content in seedlings (Halkier & Møller, 1989).

The biosynthesis of dhurrin involves two membrane‐anchored cytochrome P450 (CYP) enzymes, a membrane‐anchored CYP oxidoreductase and a soluble UGT (Knudsen et al., 2018; Laursen et al., 2015; Møller & Conn, 1979). The CYP79A1 serves as entry point of the dhurrin pathway, displaying high substrate specificity towards l‐tyrosine and catalyzing the production of (*E*)‐*p*‐hydroxyphenylacetaldoxime (Clausen et al., 2015; Halkier et al., 1995; Koch et al., 1995). Following this, (*E*)‐*p*‐hydroxyphenylacetaldoxime is converted into (*S*)‐*p*‐hydroxymandelonitrile, in a reaction catalyzed by CYP71E1 (Bak et al., 1998; Kahn et al., 1997). Finally, (*S*)‐*p*‐hydroxymandelonitrile is stabilized by glucosylation catalyzed by *Sb*UGT85B1, resulting in the formation of dhurrin (Jones et al., 1999).

The dhurrin biosynthetic pathway has been shown to organize in a complex on the cytosolic surface of the ER membrane (Laursen et al., 2021), known as a metabolon, resulting in highly efficient substrate channeling of l‐tyrosine towards dhurrin (Laursen et al., 2016). The soluble *Sb*UGT85B1 was shown to play a key function in the metabolon assembly by organizing the membrane‐anchored CYP enzymes (Laursen et al., 2016).

**Figure 1:**
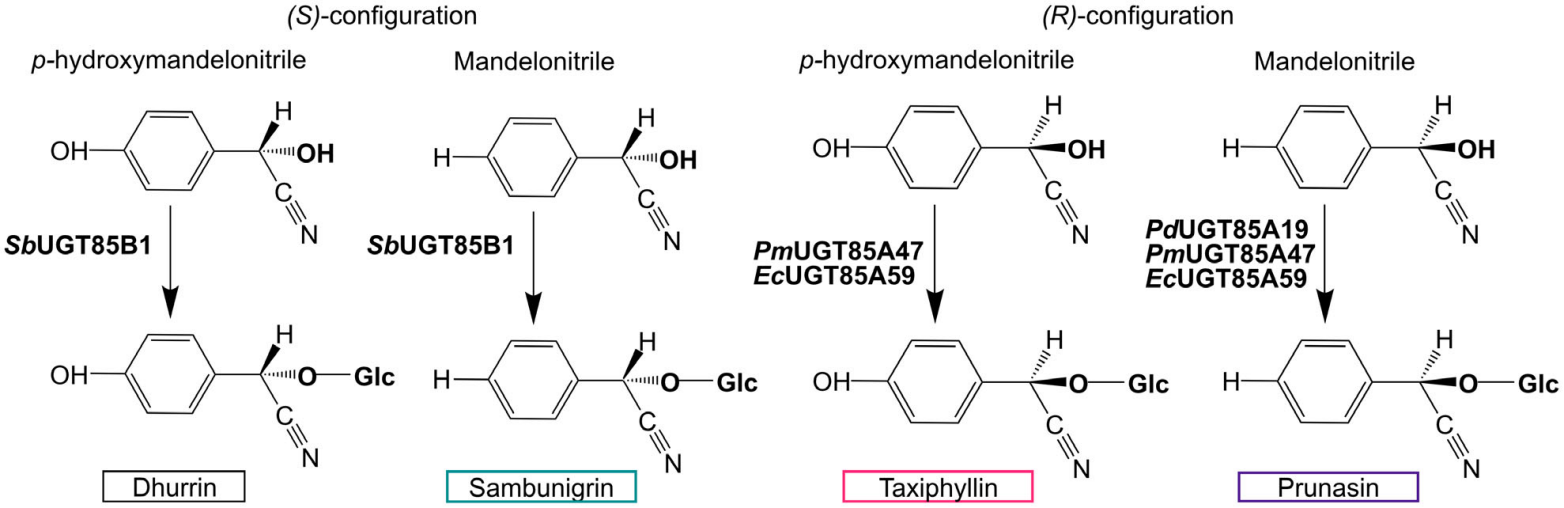
Schematic representation of the known reactions catalyzed by the UGT85 enzymes described in this paper.

*Sb*UGT85B1 has been reported to also catalyze the *in vitro* glycosylation of (*S*)‐mandelonitrile to sambunigrin displaying stereo‐selectivity towards the (*S*)‐configuration of cyanohydrins (Hansen et al., 2003) (Figure 1, left). In contrast, the UGTs catalyzing the biosynthesis of prunasin and amygdalin from l‐phenylalanine are specific to the (*R*)‐configuration of the cyanohydrins. This is the case of UGT85A19 from almond (*Prunus dulcis*, *Pd*UGT85A19) (Franks et al., 2008), UGT85A47 from Japanese apricot (*Prunus mume*, *Pm*UGT85A47) (Yamaguchi & Asano, 2018), and UGT85A59 from *Eucalyptus cladocalyx* (*Ec*UGT85A59) (Hansen et al., 2018) (Figure 1, right).

Given that UGTs play a key role in the biosynthesis of almost all major classes of natural products, there is a pressing demand for understanding the structural background determining substrate specificity and protein–protein interaction patterns. Despite the UGT85 family being present in high copy number in many land plant species and various UGT85 family members being functionally characterized (Wilson & Tian, 2019), the only member of the UGT85 family for which a structure has been determined is UGT85H2 from *Medicago truncatula* catalyzing glycosylation of flavonoids (Li et al., 2007). Accordingly, there is no available crystal structure for any UGT involved in biosynthesis of cyanogenic glucosides, despite *Sb*UGT85B1 being well characterized and known for more than 40 years. This has challenged the elucidation of the structural basis governing substrate specificity. However, residues involved in catalysis in *Sb*UGT85B1 were predicted by molecular modeling based on hydrophobic cluster analysis combined with ligand docking (Thorsøe et al., 2005).

In the current study, we aimed to structurally characterize *Sb*UGT85B1 using X‐ray crystallography and to investigate its acceptor substrate specificity and stereo‐selectivity by structure‐guided mutational analysis. We compared these findings to two other UGTs involved in the biosynthesis of aromatic cyanogenic glucosides. These studies enabled identification of key amino acids determining substrate and stereo‐specificity of aromatic cyanogenic glucoside biosynthesis.

## RESULTS AND DISCUSSION

### Structural insight into *Sb*UGT85B1 in complex with UDP and *p*‐hydroxymandelonitrile

To elucidate the structure–function relationships governing *Sb*UGT85B1 specificity, we solved the X‐ray crystal structures of *Sb*UGT85B1 bound to UDP (1.43 Å) (PDB‐ID: 7ZER) and to UDP + *p*‐hydroxymandelonitrile (1.50 Å) (PBD‐ID: 7ZF0). *Sb*UGT85B1 was expressed in *Escherichia coli* and purified to homogeneity obtaining approximately 150 mg purified protein per liter of bacterial culture (Figure S1). Crystals were developed by the sitting drop method and the structure was solved *via* molecular replacement using *Mt*UGT85H2 (Li et al., 2007).

**Figure 2:**
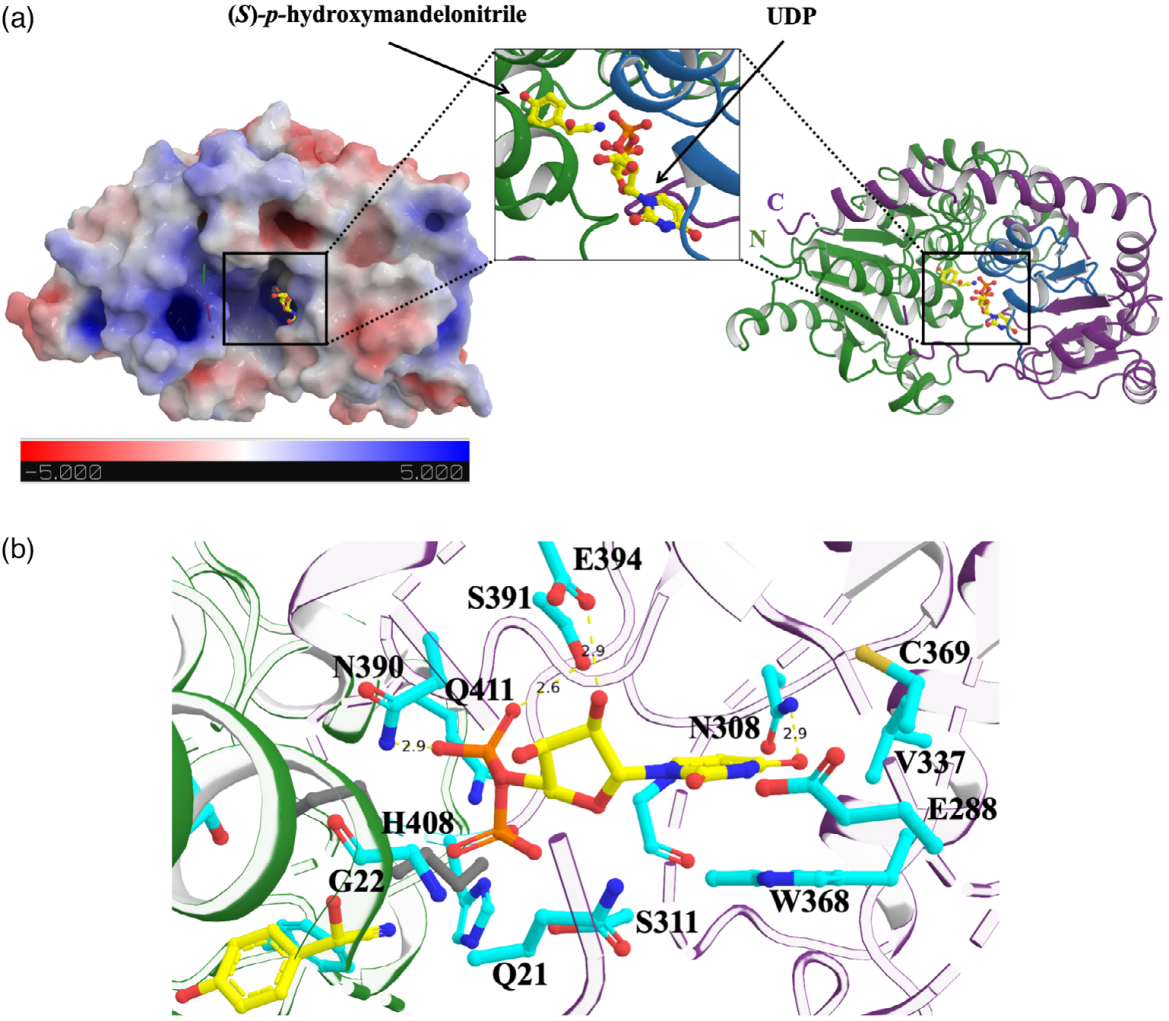
(a) Electrostatic surface view (left) and ribbon representation (right) of the *Sb*UGT85B1 crystal structure showing (*S*)‐*p*‐hydroxymandelonitrile and UDP in the active site with a narrow entrance. Ribbon representation with the N‐terminal domain shown in forest green and the C‐terminal domain in purple. The PSPG motif is shown in skyblue. (b) UDP in the active site of *Sb*UGT85B1. Hydrogen bonds are shown with dashed yellow lines. Ethylene glycol molecules from the crystallization buffer are shown as gray sticks.

The structures reveal the characteristic UDP‐glycosyltransferase GT‐B fold found in all known plant UGTs (Lairson et al., 2008) with two Rossman domains forming a narrow catalytic cleft, where the N‐terminal domain predominantly interacts with *p*‐hydroxymandelonitrile, while the C‐terminal domain predominantly interacts with UDP (Figure 2). The two structures are highly similar (root mean square deviation [RMSD] = 0.17 Å over all 6148 atoms), thus not indicating significant structural changes upon binding of *p*‐hydroxymandelonitrile (Figure S2a). The His23–Asp130 motif corresponding to the highly conserved catalytic dyad of plant UGTs was found near the glycosylation site of the substrate (Figure S2b) (Gutmann & Nidetzky, 2013). This is in accordance with the reported reaction mechanism, S_N_2‐like nucleophilic substitution, which is facilitated by the histidine as the catalytic base deprotonating the hydroxyl oxygen of the acceptor substrate and by the aspartate stabilizing the charge after proton abstraction (Brazier‐Hicks et al., 2007; Lairson et al., 2008; Teze et al., 2021).

The UDP binding site is characterized by the presence of the highly conserved family‐defining Plant Secondary Product Glycosyltransferase (PSPG) motif (Osmani et al., 2009), and consists of residues W368, C369, Q371, H386, N390, S391, E394, and Q411, as well as residues Q21, E288, N308, S311, and V337. Coordination of S391 with the pyrophosphate group confirms a previous hypothesis of S391 to stabilize binding of UDP‐glucose (Thorsøe et al., 2005). In contrast, R201, also previously hypothesized to stabilize binding of UDP‐glucose (Thorsøe et al., 2005), is not in the active site, and is located 22 Å from UDP. The binding mode of UDP in the sugar donor active site is identical in the two structures, and thus not affected by the presence of *p*‐hydroxymandelonitrile in the sugar acceptor binding site (Figures 2b and S2).

The electron density in the acceptor binding site was easily interpretable and clearly indicates the bound *p*‐hydroxymandelonitrile to be in the (*S*)‐configuration, even though *Sb*UGT85B1 was co‐crystallized with a racemic mixture of both the (*R*)‐ and (*S*)‐configurations (Figure S3). This is in accordance with reported higher *in vitro* activity towards (*S*)‐*p*‐hydroxymandelonitrile compared with (*R*)‐*p*‐hydroxymandelonitrile (Hansen et al., 2003). *p*‐Hydroxymandelonitrile was found in hydrogen‐bonding distance to the catalytic histidine H23 (2.7 Å), glutamine Q225 (3.0 Å), and a water molecule (2.9 Å) (Figure 4a). There is a hydrogen bond between the amino group of the Q225 side chain and the *p*‐hydroxyl group of the acceptor. Additionally, the acceptor binding pocket is lined with five hydrophobic residues (F18, V90, V94, V132, and F208). The glutamic acid E410, previously hypothesized to interact with the substrate (Thorsøe et al., 2005), although lining up with the active site, is at 5.4 Å from the closest atom in the substrate (the nitrogen of *p*‐hydroxymandelonitrile). However, it is not possible to exclude that E410 could move closer to the substrate and play a role during catalysis.

The hydrophobic patch composed of residues 156–167 previously proposed to be involved in interaction with CYP enzymes (Thorsøe et al., 2005) is buried in the crystal structure of the protein, making these residues unavailable for interactions with other proteins (Figure S4). Furthermore, based on the crystal structure of *Sb*UGT85B1 it was not possible to identify other candidate surfaces that might mediate the UGT–CYP interaction. Therefore, additional studies are needed to unveil the structural determinants for UGT–CYP interaction and metabolon stabilization.

Additionally, we compared both structures with the Alphafold model of UGT85B1 (ID: AF‐Q9SBL1‐F1) which shows very high confidence for most of the residues (pLDDT > 90) and quite accurate RMSD scores (0.63 Å and 0.62 Å for 7ZF0 and 7ZER, respectively). However, there are many residues, predominantly on the protein surface (some in the very high‐confidence region) showing significant differences in the side chain orientations compared to the X‐ray structures.

### Engineering of *Sb*UGT85B1

**Figure 3:**
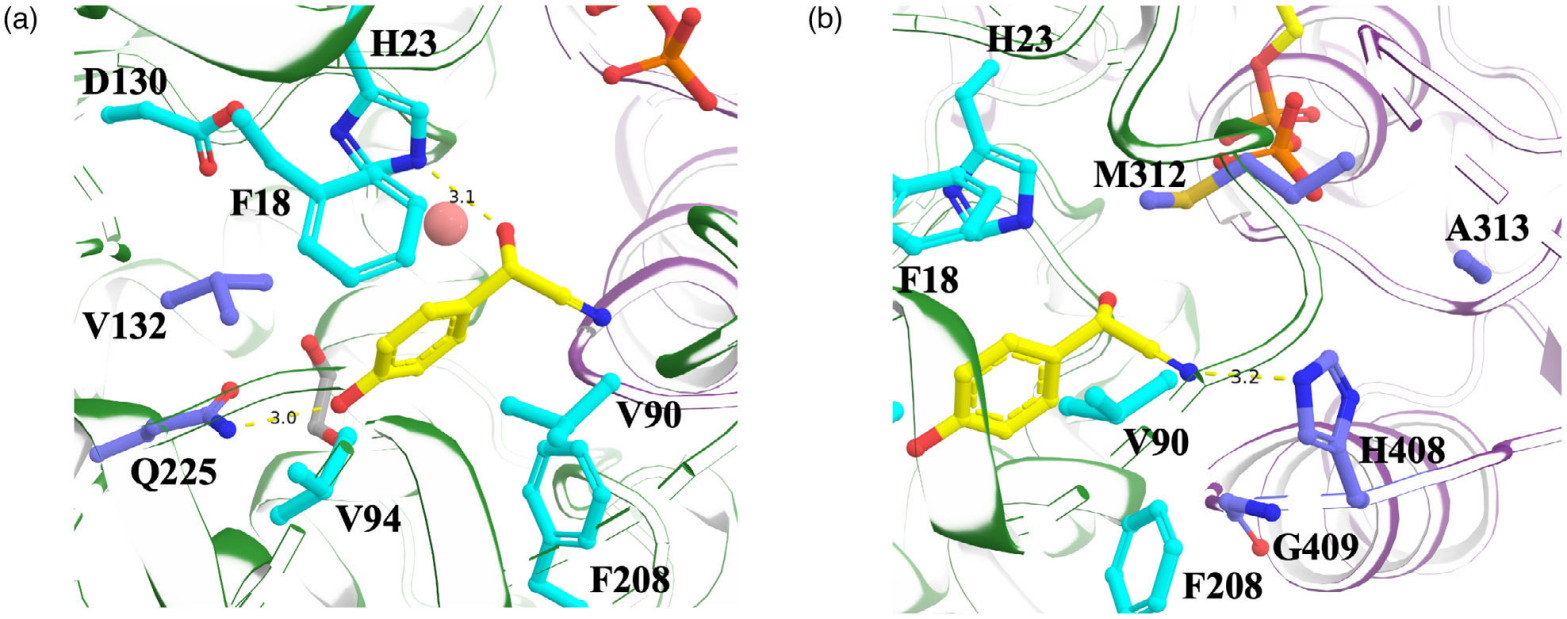
(*S*)‐*p*‐Hydroxymandelonitrile in the active site of *Sb*UGT85B1. Selected residues for mutagenesis to shift acceptor substrate specificity from *p*‐hydroxymandelonitrile to mandelonitrile (a) and stereo‐specificity from the (*S*)‐ to the (*R*)‐isomer (b) are shown in slate blue. Ethylene glycol is shown as gray sticks and water as a salmon‐colored sphere.

To identify key amino acids controlling the preference for *p*‐hydroxy‐substituted acceptor, we designed four mutants that would abolish a hydrogen bond between the *p*‐hydroxyl group and a conserved glutamine (Q225E/W/F/M). In addition, we designed two double mutants where a nearby valine is substituted with an alanine, in order to create an opening for the introduced bulky amino acids (V132A/Q225W and V132A/Q225F) (Figure 3a).

The activities of these mutants were tested towards racemic mixtures comprising the (*R*)‐ and (*S*)‐configurations of both mandelonitrile and (*p*)‐hydroxymandelonitrile for the production of prunasin and sambunigrin or dhurrin and taxiphyllin, respectively. The concentration of substrate used is expected to be above the K_m_ for UGT85B1, since this enzyme showed K_m_ values ranging from 0.1 to 0.3 mm for all the natural substrates previously tested (Hansen et al., 2003). Thus, the enzymatic reactions are expected to occur at near optimal conditions suitable for relative comparison between mutants.

**Figure 4:**
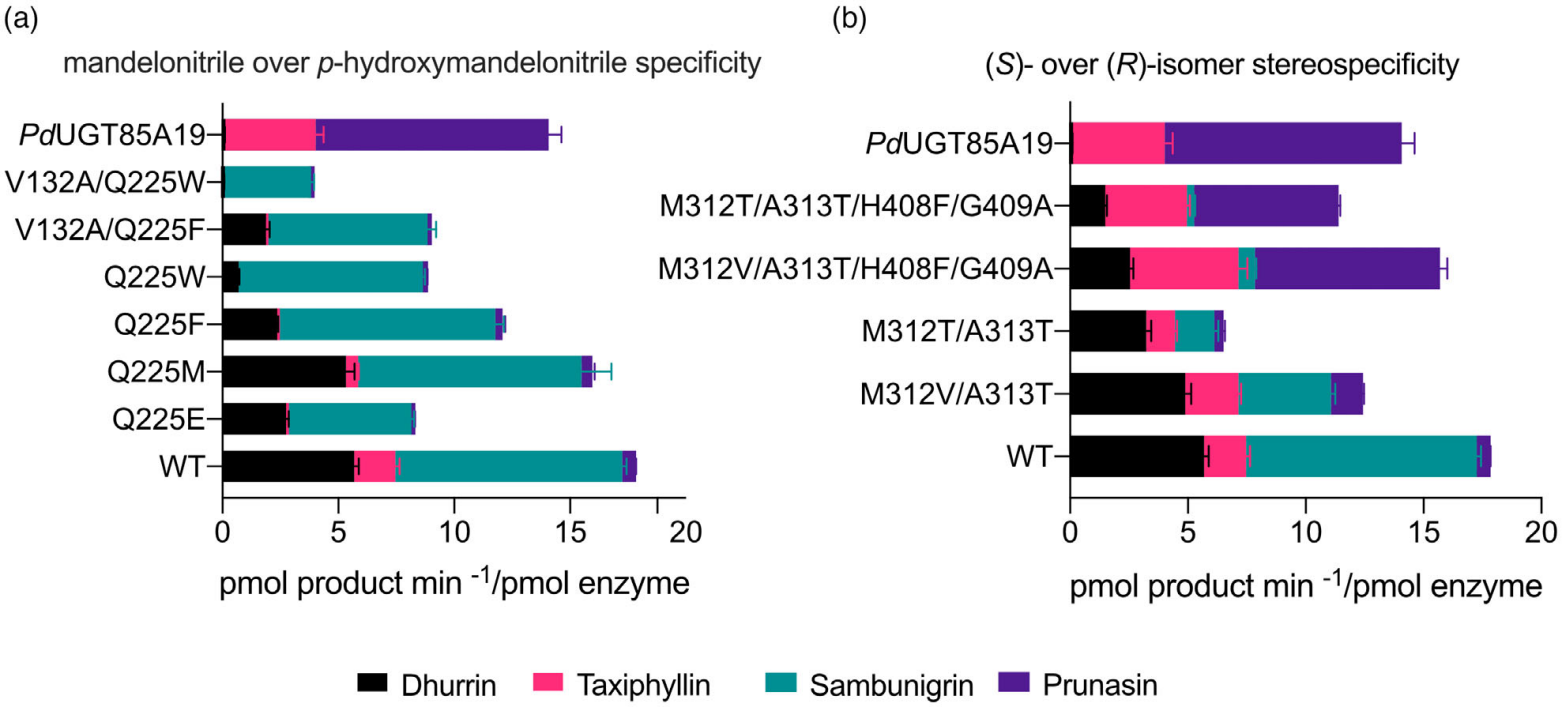
Activity assay on *Sb*UGT85B1 mutants. The activity assays were performed by using as substrate racemic mixtures of either *p*‐hydroxymandelonitrile or mandelonitrile and the amounts of products generated measured by LC‐MS/MS. The catalytic activity of the *Sb*UGT85B1 mutants designed to have (a) mandelonitrile specificity or (b) stereo‐specificity towards the (*R*)‐isomer of the substrates was measured in *E*. *coli* cell lysate. Wild‐type *Sb*UGT85B1 and *Pd*UGT85A19 are included as controls. Significance values are reported in Table S1.

Although the *Sb*UGT85B1 structure revealed the sole presence of the (*S*)‐*p*‐hydroxymandelonitrile in the active site of the protein, our data demonstrated that this enzyme is also able to catalyze the conversion of the (*R*)‐isomer into taxiphyllin (Figure 4a). We also investigated the activities with the four substrates for *Pd*UGT85A19, which glucosylate (*R*)‐mandelonitrile in almond (Franks et al., 2008). The data revealed that *Pd*UGT85A19 is also able to catalyze the formation of taxiphyllin by utilizing (*R*)‐*p*‐hydroxymandelonitrile as substrate, providing evidence for such a reaction for this UGT.

The analysis of the mutants revealed that their activity towards either (*R*)‐ or (*S*)‐*p*‐hydroxymandelonitrile was notably decreased, as the amounts of dhurrin and taxiphyllin produced dropped significantly, with the V132A/Q225W mutant showing a complete loss of activity towards dhurrin. Importantly, the mutants still retain the ability to convert (*S*)‐mandelonitrile into sambunigrin, although the V132A/Q225W mutant showed a decrease in the amount of the sambunigrin produced, as compared to the wild‐type *Sb*UGT85B1 (Figure 4a).

To identify key amino acids controlling the preference towards the (*S*)‐ over the (*R*)‐isomer, active site residues within 6 Å of the nitrile group were inspected (Figure 3b), considering both the (*S*)‐ and (*R*)‐forms of the acceptor, although only the (*S*)‐isomer was found in the crystal structure. The residues were compared to the corresponding residues in the (*R*)‐specific *Pd*UGT85A19 and *Pm*UGT85A47, which have 46% sequence identity with *Sb*UGT85B1 (Figure S5). We designed two double mutants (M312T/A313T and M312V/A313T) and two quadruple mutants (M312T/A313T/H408F/G409A and M312V/A313T/H408F/G409A) based on structural analysis and multiple sequence alignment with the (*R*)‐mandelonitrile‐specific UGTs. We hypothesized that methionine at position 312 might sterically hinder a productive binding orientation of the (*R*)‐isomer, since this position is occupied by a much smaller residue (threonine or valine) in the (*R*)‐specific UGTs. To further mimic the (*R*)‐specific UGTs, we also substituted the neighbor alanine with a threonine. In addition, in order to reduce the space close to the nitrile in the (*S*)‐configuration and thereby possibly reduce the affinity for this isomer, we substituted H408 and G409 with the corresponding amino acids in the (*R*)‐specific UGTs, phenylalanine and alanine, respectively.

The results of the activity assay presented in Figure 4b clearly show that all the tested mutants displayed a reduced ability to utilize the (*S*)‐isomer of both substrates for the production of dhurrin and sambunigrin. However, the two double mutants (M312T/A313T and M312V/A313T) did not show any increased ability to glucosylate the (*R*)‐isomer of both *p*‐hydroxymandelonitrile and mandelonitrile to form taxiphyllin and prunasin, respectively. Different is the case for the quadruple mutants, for which the reduction of the amount of dhurrin and sambunigrin produced (0.74‐ and 0.97‐fold change in dhurrin and sambunigrin levels for M312V/A313T/H408F/G409A compared to the wild‐type enzyme) occurs alongside with a large increase of taxiphyllin and prunasin production (1.94‐ and 10.2‐fold change in taxiphyllin and prunasin levels for M312V/A313T/H408F/G409A compared to the wild‐type enzyme).

### Stereo‐specificity among UGT85 enzymes

The activity data revealed that UGT85s involved in cyanogenic glucoside biosynthesis display high stereo‐specificity with preference for either the (*S*)‐ or the (*R*)‐cyanohydrin configuration (Figure 4). *Sb*UGT85B1 has a clear preference for the (*S*)‐configuration of *p*‐hydroxymandelonitrile. In the plant and upon tissue disruption, dhurrin comes in contact with an otherwise spatially separated beta‐glucosidase, dhurrinase (Morant et al., 2008), with a high specificity towards the (*S*)‐configuration of the substrate as evident from hydrolysis of dhurrin but not taxiphyllin (Hösel et al., 1987). This suggests that stereo‐specificity co‐evolves in both biosynthetic and catabolic pathways.

**Figure 5:**
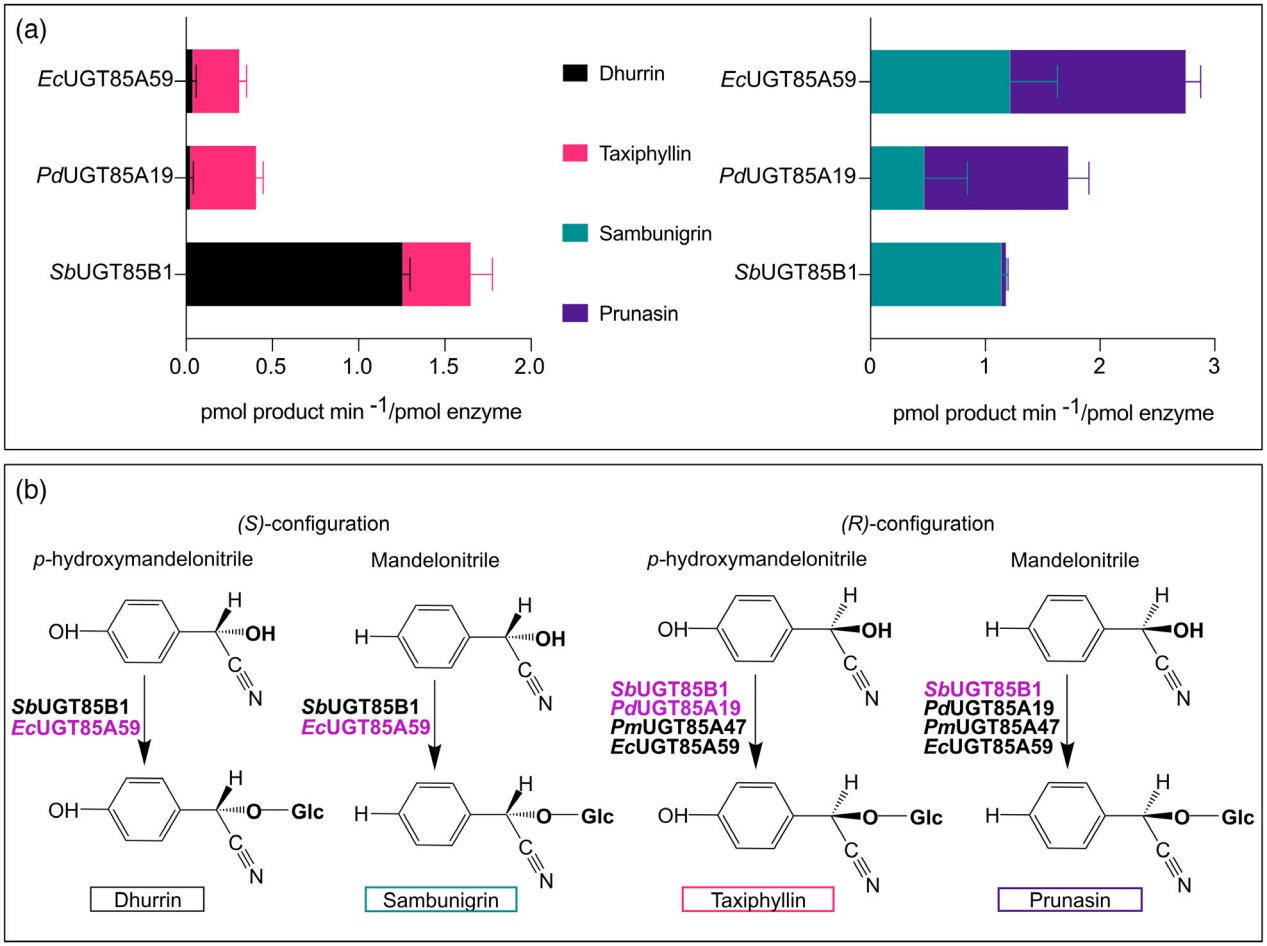
Comparative functional analysis on *Sb*UGT85B1, *Pd*UGT85A19, and *Ec*UGT85A59 (a) and updated schematic representation of the reactions catalyzed (b) by the UGTs described in this paper.

While *Sb*UGT85B1 mostly produced dhurrin using *p*‐hydroxymandelonitrile as substrate, some taxiphyllin was also produced (Figures 4 and 5). Moreover, it was also able to catalyze glucosylation of mandelonitrile mainly resulting in sambunigrin, but also trace amounts of prunasin. In contrast, *Pd*UGT85A19 was highly specific towards the (*R*)‐configuration, as demonstrated by production of prunasin and taxiphyllin but only trace amounts of sambunigrin and no dhurrin (Figure 5).

In order to evaluate the conservation of stereo‐specificity among UGT85s involved in aromatic cyanogenic glucoside biosynthesis, we also included *Ec*UGT85A59 from *E*. *cladocalyx*, which is involved in prunasin production (Hansen et al., 2018). Similar to *Pd*UGT85A19, *Ec*UGT85A59 displayed a preference for (*R*)‐mandelonitrile but also produced trace amounts of sambunigrin. The same pattern was observed when *p*‐hydroxymandelonitrile was used as substrate (Figure 5a). Indeed, besides the main activity for production of prunasin, *Ec*UGT85A59 is able to convert *p*‐hydroxymandelonitrile into taxiphyllin and trace amounts of dhurrin. The less stringent stereo‐specificity of *Ec*UGT85A59 is likely due to the presence of a valine in position 312 instead of a threonine, as in *Pd*UGT85A19 (Figure S6). This hypothesis is supported by the observation that the *Sb*UGT85B1 quadruple mutant carrying the M312V mutation (M312V/A313T/H408F/G409A) shows a less pronounced stereo‐selectivity compared to the other quadruple mutant carrying a threonine in the same position (M312T/A313T/H408F/G409A) (Figure 4). Thus, these results allow us to pinpoint factors that drive stereo‐specificities in the cyanogenic glucoside biosynthetic enzymes and expand the reaction portfolio for the UGTs involved in the production of the cyanogenic glucosides (Figure 5b). Interestingly, some cyanogenic *Eucalyptus* species also produce sambunigrin (Gleadow et al., 2008). Comparative studies of different UGTs involved in cyanogenic glucoside biosynthesis in *Eucalyptus* might provide further understanding of factors important for stereo‐specificity.

The fact that the tested UGTs are able to utilize both the (*S*)‐ and the (*R*)‐configuration of the substrates *in vitro*, although there is no evidence for such reactions *in vivo*, confirms the dhurrin metabolon is formed and substrate is channeled in *S*. *bicolor* (Laursen et al., 2016) and provides an indirect demonstration of metabolic channeling in the biosynthesis of prunasin. Indeed, if we exclude the substrate channeling, the (*R*)‐mandelonitrile would spontaneously epimerize, and both the (*S*)‐ and (*R*)‐configurations would be available as sugar acceptors for *Pd*UGT85A19 or *Ec*UGT85A59. However, only prunasin is detected in almond and *E*. *cladocalyx*, pointing towards efficient substrate channeling and metabolon formation.

## CONCLUSIONS

Glycosylation is an important step for the biosynthesis of natural products, conferring them stability and enormously increasing their diversity of structure and function. The plant enzymes catalyzing this reaction constitute a large family and have enormously evolved to accommodate different sugar acceptors, with high stereo‐specificity.

In this study, we report the X‐ray structure of *Sb*UGT85B1, responsible for the production of the cyanogenic glucoside dhurrin in *S. bicolor*, and the structure‐driven identification of key amino acids determining substrate and stereo‐specificity of aromatic cyanogenic glucoside biosynthesis. Furthermore, performing a comparative functional analysis with two other UGTs involved in the biosynthesis of cyanogenic glucosides, *Pd*UGT85A19 and *Ec*UGT85A59, we expanded the range of known reactions catalyzed by these enzymes using mandelonitrile and *p*‐hydroxymandelonitrile as sugar acceptors (Figure 5b).

In perspective, the availability of a high‐resolution structure of *Sb*UGT85B1 opens the way to several studies and applications. Firstly, it will lead to a deeper understanding of the molecular factors that govern biosynthesis of dhurrin and participation of *Sb*UGT85B1 in metabolon assembly. Indeed, although in this study we did not identify surfaces that might mediate the UGT–CYP interaction, the availability of the structure will enable detailed interaction studies at the molecular level. A possible approach might be the crosslinking‐mediated identification of UGT–CYP interacting surfaces and functional assays on specific UGT mutants in the presence of CYP. Furthermore, *Sb*UGT85B1 has been previously proved to be a highly stable enzyme, able to retain more than 50% activity upon storage in glycerol at room temperature for 180 days (Knudsen et al., 2020). This feature, combined with the rational design of substrate preference, points towards the use of *Sb*UGT85B1 as an excellent candidate for biotechnological purposes for tailored glycosylation of a broad range of natural products. Finally, the high resolution of the UDP–sugar binding site may enable engineering of sugar donor preference for the production of new‐to‐nature compounds with tailored properties.

## MATERIALS AND METHODS

### Recombinant proteins production in *E. coli*

The codon‐optimized *S. bicolor UGT85B1* (NCBI reference sequence: Q9SBL1.1 [https://www.ncbi.nlm.nih.gov/protein/Q9SBL1.1]), subcloned into the pET30a vector (GenScript Biotech), was expressed in the *E. coli* strain NiCo21 (DE3) (New England Biolabs, Ipswich, MA, USA). A 200‐ml starter culture of terrific broth (TB) supplemented with 50 μg ml^−1^ kanamycin was grown overnight at 37°C and 250 rpm. The starter culture was diluted into 2 L of TB supplemented with kanamycin in a 2‐L bench‐top fermenter (Biostat®A, Sartorius) operated at 37°C and 80% O_2_ saturation. The pH of the medium was controlled at 7.4 by using 16% NaOH and 20% H_2_SO_4_. The dissolved oxygen (DO) was maintained at 40% by a cascade controller of agitation speed and air flow rate. Expression was induced when the culture reached OD_600nm_ = 1 by the addition of isopropyl β‐d‐1‐thiogalactopyranoside (IPTG) to a final concentration of 1 mm. Cells were harvested after expression for 16 h at 25°C by centrifugation (5000 × ***g***, 15 min) and stored at −70°C until further use.

Codon‐optimized *Sb*UGT85B1 mutants, *Pd*UGT85A19 (NCBI reference sequence: ABV68925.1 [https://www.ncbi.nlm.nih.gov/protein/ABV68925.1]) subcloned into the pET30a vector and *Ec*UGT85A59 subcloned in pET‐28a(+)‐TEV (NCBI reference sequence: 1495988848 [https://www.ncbi.nlm.nih.gov/protein/1495988848]), were expressed in the *E. coli* strain NiCo21 (DE3). The starter culture, TB medium supplemented with 50 μg ml^−1^ kanamycin grown overnight at 37°C and 250 rpm, was diluted 1:10 into 25 ml of TB supplemented with kanamycin, in 250‐ml baffled flasks and incubated at 37°C. Protein expression was induced when the culture reached OD_600_ = 0.6 by adding 1 mm IPTG for *Sb*UGT85B1 mutants and *Pd*UGT85A19 and adding 0.5 mm IPTG for *Ec*UGT85A59. Cells were harvested after 16 h at 25°C for *Sb*UGT85B1 mutants and *Pd*UGT85A19 and at 20°C for *Ec*UGT85A59 and stored at −70°C until further use.

### Purification of the recombinant enzymes

*Sb*UGT85B1 was purified using three chromatography steps: immobilized metal ion affinity chromatography (IMAC), ion exchange chromatography (IEX), and size exclusion chromatography (SEC).

The *E. coli* cell pellets overexpressing different UGT variants carrying a tag of six histidines at the C‐terminus were re‐suspended in buffer containing 50 mm Tris–HCl pH 7.5, 100 mm NaCl, and 1 mm DTT supplemented with 1 tablet cOmplete™ protease inhibitor cocktail (Roche) per 100 ml buffer. Cells were lysed using a cell disrupter (Constant Systems Ltd., Daventry, England, UK) using a process pressure of 25 kPSI. Cell debris was sedimented by centrifugation (14 000 × ***g***, 30 min) and the supernatant was applied to a 5 ml HisTrap® HP (Cytiva), equilibrated with buffer A containing 50 mm Tris–HCl pH 7.5, 100 mm NaCl, and 1 mm DTT and operated at 5 ml min^−1^. After loading, the column was extensively washed with 10% buffer B (50 mm Tris–HCl pH 7.5, 100 mm NaCl, 1 mM DTT, and 500 mm imidazole) and the proteins were eluted by isocratic elution in 100% buffer B. The main peak fractions were pooled, and the sample was desalted in 50 mm Tris–HCl pH 7.5, 100 mm NaCl, and 1 mm DTT using a 5 ml HiTrap® Desalting Column (Cytiva).

The sample after IMAC purification was concentrated using Amicon® Ultra centrifugal filters (30 K, Merck Millipore) and loaded to a strong anion exchange column (Mono Q® 5/50 GL; Sigma‐Aldrich, St. Louis, MO, USA). Before the sample application the column was equilibrated with 95% buffer A containing 20 mm Tris–HCl pH 7.5 and 1 mm DTT and 5% buffer B consisting of 20 mm Tris–HCl pH 7.5, 1 m NaCl, and 1 mm DTT. After loading the sample, proteins were eluted with a linear gradient from 50 to 400 mm NaCl using buffer A and buffer B starting with 5% buffer B and increasing the percentage of buffer B to 40%. The flow rate was 1.5 ml min^−1^. The main UV‐absorbing peak fractions were collected, concentrated, and subjected to gel filtration chromatography using a Superdex® 200 Increase 10/300 GL column (Cytiva, Marlborough, MA, USA). The running buffer was 25 mm HEPES pH 7.0 with 50 mm NaCl and 1 mm DTT. The main peak fractions were analyzed by SDS‐PAGE (NuPAGE 4–12% Bis‐Tris, Invitrogen) and those of the highest purity were pooled and used for crystallization screens after adjusting the concentration.

*Pd*UGT85A19 was purified using IMAC followed by IEX, as described above for *Sb*UGT85B1.

For *Eu*UGT85A49 purification, cell pellets were suspended in lysis buffer containing 100 mm Tris pH 7.5, 300 mm NaCl, 1 mm MgCl_2_, 0.5 mm tris(2‐carboxyethyl)phosphine (TCEP), 0.04 lysozyme mg ml^−1^, benzonase (25 U ml^−1^; Sigma‐Aldrich), and 1 mm PMSF and lysed using a high‐pressure homogenizer at 20 kPSI (Avestin EmulsiFlex D20). The lysate was centrifuged at 9600 × ***g*** for 40 min at 4°C. Cleared lysate was filtered (0.45 μm pore size) and then applied to an equilibrated 5 ml HisTrap^®^ FF column (GE Healthcare, Chicago, IL, USA). Upon loading, the column was extensively washed with 10 column volumes (CVs) of wash buffer (50 mm NaP pH 7.5, 300 mm NaCl, 30 mm imidazole, and 0.5 mm TCEP), followed by a linear gradient of 0–7.5% elution buffer (50 mm NaP pH 7.5, 300 mm NaCl, 500 mm imidazole, and 0.5 mm TCEP) and an isocratic phase of 2 CV at 7.5%. The composition was subsequently linearly increased to 35% elution buffer, and *Ec*UGT85A59 was eluted at an isocratic phase of 35% elution buffer. Fractions containing UGT85A59 were pooled and concentrated by centrifugation using a pre‐equilibrated membrane filter (30 kDa cut‐off; Thermo Scientific). The concentrate was applied to an equilibrated HiLoad 16/60 Superdex^®^ 200 column (120 ml, GE Healthcare), and protein was eluted in 50 mm NaP pH 7.5 with 100 mm NaCl, 0.5 mm TCEP, and 10% (w/v) glycerol.

### Crystallization of *Sb*UGT85B1 with UDP and *p*‐hydroxymandelonitrile

The initial crystallization screening was carried out using a Gryphon crystallization robot in MRC 2‐well crystallization plates (Hampton Research) with MORPHEUS screen (Molecular dimensions, Newmarket, England, UK). Crystals were detected after 16–20 h in drops with 15 mg ml^−1^ protein in D2, E2, and F2 conditions using a 1:1 protein:buffer ratio (150 nl protein solution and 150 nl buffer solution). The initially obtained crystals showed poor diffraction. Thus, crystallization conditions were optimized, and well‐diffracting crystals were obtained as follows. *Sb*UGT85B1 was co‐crystallized with UDP at 293 K in sitting drops consisting of 2 μl protein solution (14 mg ml^−1^ *Sb*UGT85B1 in 25 mm HEPES pH 7.0, 50 mm NaCl, 1 mm TCEP, and 5 mm UDP) and 2 μl crystallization buffer consisting of 100 mm MES/imidazole pH 6.5, 12.5% (w/v) PEG8000 (BioUltra, Sigma, St. Louis, MO, USA), 12.5% (v/v) ethylene glycol (≥99%, Sigma), and 120 mm MORPHEUS monosaccharides mix consisting of equimolar concentrations of d‐glucose, d‐mannose, d‐galactose, l‐fucose, d‐xylose, and N‐acetyl‐d‐glucosamine (Molecular dimensions). Crystals were detected after approximately 16 h and were of the close‐to‐final size (200–300 μm). A few minutes before mounting the crystals in cryoloops, 0.4 mg *p*‐hydroxymandelonitrile dissolved in cryoprotectant ethylene glycol (40% in crystallization buffer) was added to the drop (Figure S7a).

### *Sb*UGT85B1 structure determination and data analysis

First, 270° of data were collected at 100 K at the BioMax beamline of the MAX‐IV Laboratory (Lund, Sweden) with an oscillation of 0.05°, an exposure time of 0.011 sec, and a Eiger16M detector (Figure S7b). The data were processed with XDSAPP (Kabsch et al., 2010) and the structure was solved *via* molecular replacement using UGT85H2 (Li et al., 2007) from *M. truncatula* (PDB ID:2PQ6, approximately 43% identity) as the search model and PHASER (McCoy et al., 2007) from the Phenix software package (Liebschner et al., 2019). The structures were rebuilt and refined using Phenix Refine and Coot (Emsley & Cowtan, 2004) to a final R‐work of 18.4 and 19.2 and an R‐free of 19.7 and 21.3, with 98.08% of the residues in the favored region of the Ramachandran plot with 0% outliers. The final structures were validated with MolProbity (Chen et al., 2010). Data collection and refinement statistics are presented in Table S2. Structural figures as well as *Sb*UGT85B1:*p*‐hydoxymandelonitrile interactions were created and analyzed using PyMOL software (version 2.4.2, Schrödinger, Inc., New York, NY, USA).

### *Sb*UGT85B1 mutagenesis

*Sb*UGT85B1 mutants were prepared using the QuikChange Lightning® site‐directed mutagenesis kit (Agilent, Santa Clara, CA, USA) following the manufacturer's protocol, and the mutations were confirmed by sequencing (Macrogen Europe, Amsterdam, Netherlands). The primer pairs used to introduce the selected mutations were designed using the QuikChange Primer Design tool available on the Agilent website (https://www.agilent.com/store/primerDesignProgram.jsp) and are listed in Table S3.

### Activity of *Sb*UGT85B1 mutants

Activity assays of all *Sb*UGT85B1 mutants were performed directly on the cell lysates. Cell pellets were resuspended in 4 ml buffer (50 mm Tris–HCl pH 7.5, 100 mm NaCl, and 1 mm DTT) supplemented with 1 tablet cOmplete™ protease inhibitor cocktail (Roche, Basel, Switzerland) per 100 ml, and cells were lysed using a tip sonicator. The cleared cell lysate was obtained upon sedimentation of cell debris by centrifugation (14 000 × ***g***, 30 min). The concentration of enzyme in the cell lysate was determined by densitometry upon SDS‐PAGE analysis of 5 and 10 μl of each cell lysate and definite amounts of pure *Sb*UGT85B1 (from 0.5 to 6 μg) to generate a reference plot correlating protein density to protein amount (Figure S8).

The assays were carried out in triplicate in a final volume of 60 μl, and the assay reaction consisted of 0.62 μm enzyme, 600 μm UDP‐glucose, and 600 μm of racemic mixtures of either *p*‐hydroxymandelonitrile (chemically synthesized) or mandelonitrile (Sigma‐Aldrich, St. Louis, MO, USA) in buffer (50 mm Tris–HCl pH 7.5, 100 mm NaCl, and 1 mM DTT). The reaction was carried out at 30°C for 30 min and terminated by addition of 150 μl of ice‐cold MeOH. Samples were filtered (MultiScreen‐IP Filter Plate, 0.45 μm, Millipore), dried in a MiniVac Speed Vacuum Centrifuge at 30°C, and stored at −70°C until further analysis.

### Quantification of cyanogenic glucosides by LC‐MS/MS

Samples were resuspended in 85 μl of resuspension solution (10% MeOH + 0.1% formic acid in water), filtered (MultiScreen‐IP Filter Plate, 0.22 μm, Millipore), and further diluted 10 times prior to analysis by liquid chromatography coupled to tandem mass spectrometry (LC‐MS/MS). The analytical method was modified from Brimer and Dalgaard (Brimer & Dalgaard, 1984) and parameters were adjusted to allow for baseline separation of the isomer pairs and optimized for analysis by LC‐MS/MS. Briefly, chromatography was performed on a 1290 Infinity II UHPLC system (Agilent Technologies). Separation was achieved on a ZORBAX RRHD SB‐C8 column (1.8 μm, 2.1 × 100 mm, 80 Å, Agilent Technologies). Formic acid (0.05%, v/v) in water and methanol were employed as mobile phases A and B, respectively. The elution profile was: 0–2.5 min, 10% B; 2.5–8.0 min, 10–15% B; 8.0–9.0 min, 15–35% B; 9.0–9.5 min, 35–98% B; 9.5–10.5 min, 98% B; 10.5–10.6 min, 98–10% B; and 10.6–12.0 min, 10% B. The mobile phase flow rate was 400 μl min^−1^. The column temperature was maintained at 40°C. The LC system was coupled to an Ultivo Triplequadrupole mass spectrometer (Agilent Technologies) equipped with a Jetstream electrospray ion source operated in positive ion mode. The ionization parameters were optimized by infusion experiments with pure authentic standards. The ion spray voltage was set to 4000 V. The dry gas temperature was set to 325°C and the dry gas flow rate to 13 L min^−1^. The sheath gas temperature was set to 400°C and the sheath gas flow rate to 12 L min^−1^. The nebulizing gas pressure was set to 60 psi. Nitrogen was used as dry gas, nebulizing gas, and collision gas. Multiple reaction monitoring (MRM) was used to monitor precursor ion → fragment ion transitions. MRM transitions were determined by direct infusion experiments of reference standards. Detailed values for mass transitions can be found in Table S4. Both Q1 and Q3 quadrupoles were maintained at unit resolution. Ionization efficiency and linearity of ionization was monitored by analyzing dilution series ranging from 2.4 to 10 000 nm that were also used for quantification. The lower limit of detection for all four cyanogenic glucosides was <2.4 nm. The separation of the standard isomer pairs taxiphyllin–dhurrin and prunasin–sambunigrin is shown in Figure S9. All analyzed cyanogenic glucosides showed non‐linear ionization behavior. The non‐linear response was addressed using quadratic functions for quantification. Mass Hunter Quantitation Analysis for QQQ software (Version 10.1, Agilent Technologies) was used for data processing and metabolite quantification.

The standards used for the identification of dhurrin, taxiphyllin, prunasin, and sambunigrin in reaction mixtures by LC‐MS/MS were chemically synthesized in our lab according to modified procedures described elsewhere (Møller et al., 2016).

### Chemical synthesis of *p*‐hydroxymandelonitrile

Following a new protocol, *p*‐hydroxymandelonitrile was directly prepared from *p*‐hydroxybenzaldehyde based on the addition of trimethylsilyl cyanide (TMS‐CN) to the aldehyde function using a TMS‐CN/lithium perchlorate (LiClO_4_) system (Azizi & Saidi, 2003) and subsequent acidic treatment (Gassman & Talley, 2003) to afford a pure epimeric mixture of 4‐hydroxymandelonitrile (E/Z = 4:5) in quantitative yield. For details, please refer to the supporting material and methods in the supporting information file.

## AUTHOR CONTRIBUTIONS

RDG, NP, FF, TL, and DHW participated in the study design. RDG, NP, and CCH performed the production and purification of the enzymes. NP and FF solved the *Sb*UGT85b1 structure and designed the mutants. RDG produced the mutants and performed the activity assays. MSM synthesized the cyanogenic glucosides and the *p*‐hydroxymandelonitrile. BMdS and CC identified and quantified the products of the activity assay. RDG, NP, TL, and DHW drafted and edited the manuscript. All the authors discussed the results and revised and commented on the final manuscript.

## CONFLICT OF INTEREST

The authors declare no conflicts of interest.

## Supporting information


**Figure S1**. Simplified workflow of *Sb*UGT81B1 production. Left panel, SDS‐PAGE analysis of equal amounts of total bacterial cell lysates from cells grown in the absence or presence of IPTG. Middle panel, SDS‐PAGE analysis of samples representative from each step from IMAC purification. Right panel, SDS‐PAGE analysis of *Sb*UGT85B1 before and after SEC purification.
**Figure S2**. (a) Comparison of the two *Sb*UGT85B1 crystal structures with UDP (cyan) and (*S*)‐*p*‐hydroxymandelonitrile (green) bound in the active site, respectively. The two structures are very similar. (b) The His‐Asp catalytic dyad in the active site of UGT85B1 close to (*S*)‐*p*‐hydroxymandelonitrile. Hydrogen bonds are shown with dashed yellow lines and distances are shown in angstrom (Å).
**Figure S3**. Difference electron density before modeling the (*S*)‐*p*‐hydroxymandelonitrile in the active site of *Sb*UGT85B1
**Figure S4**. Cartoon (a) and surface view (b) (same orientation) of the *Sb*UGT85B1 structure. The hydrophobic cluster (residues 156–167) in the structure of *Sb*UGT85B1 is shown in cyan.
**Figure S5**. Sequence alignment on which the mutant design for altered stereo‐specificity was based. The red rectangle shows the residues selected for mutagenesis.
**Figure S6**. *Sb*UGT85B1, *Pd*UGT85A19, and *Ec*UGT85A59 sequence alignment. The red rectangle depicts the residues particularly important for the enzyme's stereo‐specificity.
**Figure S7**. The *p*‐hydroxymandelonitrile‐soaked crystals of *Sb*UGT85B1 (a) and a diffraction image (b).
**Figure S8**. SDS‐PAGE analysis of 5 and 10 μl of cell lysate from *E. coli* expressing *Sb*UGT85B1 mutants and definite amounts of pure *Sb*UGT85B1 (from 0.5 to 6 μg) used to generate a reference plot correlating protein density to protein amount.
**Figure S9**. LC‐MS/MS separation of the standard isomer pairs taxiphyllin–dhurrin (a) and prunasin–sambunigrin (b).
**Table S1**. Results of two‐way ANOVA with Dunnett's multiple comparison test, performed on activity assay data reported in Figure 4.
**Table S2**. Data collection and refinement statistics of *Sb*UGT85B1 structures.
**Table S3**. List of primers used to introduce the indicated mutations in the *SbUGT85B1* gene. The primers were designed using the QuikChange Primer Design tool available on the Agilent website (https://www.agilent.com/store/primerDesignProgram.jsp).
**Table S4**. MRM transitions for cyanogenic glucosides quantified by LC‐MS/MS.

## Data Availability

The structures presented in this paper have been deposited in the Protein Data Bank (PDB) with the following accession codes: 7ZER and 7ZF0.
