# Supplementary material for: Structure‐guided engineering of key amino acids in UGT85B1 controlling substrate and stereo‐specificity in aromatic cyanogenic glucoside biosynthesis
Source: Plant J. 2022 Aug 3;111(6):1539–49. doi: 10.1111/tpj.15904 (PMC9545476; doi:10.1111/tpj.15904)
Supplement: Supplementary file 1 — Figure S1. Simplified workflow of SbUGT81B1 production. Left panel, SDS‐PAGE analysis of equal amounts of total bacterial cell lysates from cells grown in the absence or presence of IPTG. Middle panel, SDS‐PAGE analysis of samples representative from each step from IMAC purification. Right panel, SDS‐PAGE analysis of SbUGT85B1 before and after SEC purification. Figure S2. (a) Comparison of the two SbUGT85B1 crystal structures with UDP (cyan) and (S)‐p‐hydroxymandelonitrile (green) bound in the active site, respectively. The two structures are very similar. (b) The His‐Asp catalytic dyad in the active site of UGT85B1 close to (S)‐p‐hydroxymandelonitrile. Hydrogen bonds are shown with dashed yellow lines and distances are shown in angstrom (Å). Figure S3. Difference electron density before modeling the (S)‐p‐hydroxymandelonitrile in the active site of SbUGT85B1 Figure S4. Cartoon (a) and surface view (b) (same orientation) of the SbUGT85B1 structure. The hydrophobic cluster (residues 156–167) in the structure of SbUGT85B1 is shown in cyan. Figure S5. Sequence alignment on which the mutant design for altered stereo‐specificity was based. The red rectangle shows the residues selected for mutagenesis. Figure S6. SbUGT85B1, PdUGT85A19, and EcUGT85A59 sequence alignment. The red rectangle depicts the residues particularly important for the enzyme's stereo‐specificity. Figure S7. The p‐hydroxymandelonitrile‐soaked crystals of SbUGT85B1 (a) and a diffraction image (b). Figure S8. SDS‐PAGE analysis of 5 and 10 μl of cell lysate from E. coli expressing SbUGT85B1 mutants and definite amounts of pure SbUGT85B1 (from 0.5 to 6 μg) used to generate a reference plot correlating protein density to protein amount. Figure S9. LC‐MS/MS separation of the standard isomer pairs taxiphyllin–dhurrin (a) and prunasin–sambunigrin (b). Table S1. Results of two‐way ANOVA with Dunnett's multiple comparison test, performed on activity assay data reported in Figure 4. Table S2. Data collection and [file TPJ-111-1539-s001.docx]

Supporting information to:

**Structure guided engineering of key amino acids in UGT85B1 controlling substrate and stereo specificities in aromatic cyanogenic glucoside biosynthesis**

Rita Del Giudice^1,$^, Natalia Putkaradze^2,$^, Bruna Marques dos Santos^1^, Cecilie Cetti Hansen^1^, Christoph Crocoll^1^, Mohammed Saddik Motawia^1^, Folmer Fredslund^2^, Tomas Laursen^1,*^, Ditte Hededam Welner^2,*^

^$^These authors contributed equally to the work

*Corresponding authors: Tomas Laursen, tola@plen.ku.dk; Ditte Hededam Welner, diwel@biosustain.dtu.dk

**Affiliations**

^1^ Plant Biochemistry, Department of Plant and Environmental Sciences, University of Copenhagen, Thorvaldsensvej 40, DK-1871 Copenhagen, Denmark

^2^The Novo Nordisk Foundation Center for Biosustainability, Technical University of Denmark, Kemitorvet 220, DK-2800 Kgs. Lyngby, Denmark

Data included in this file:

Supporting figures and legends (Figure S1-S9)

Supporting tables (Table S1-S4)

Supporting Materials and Methods


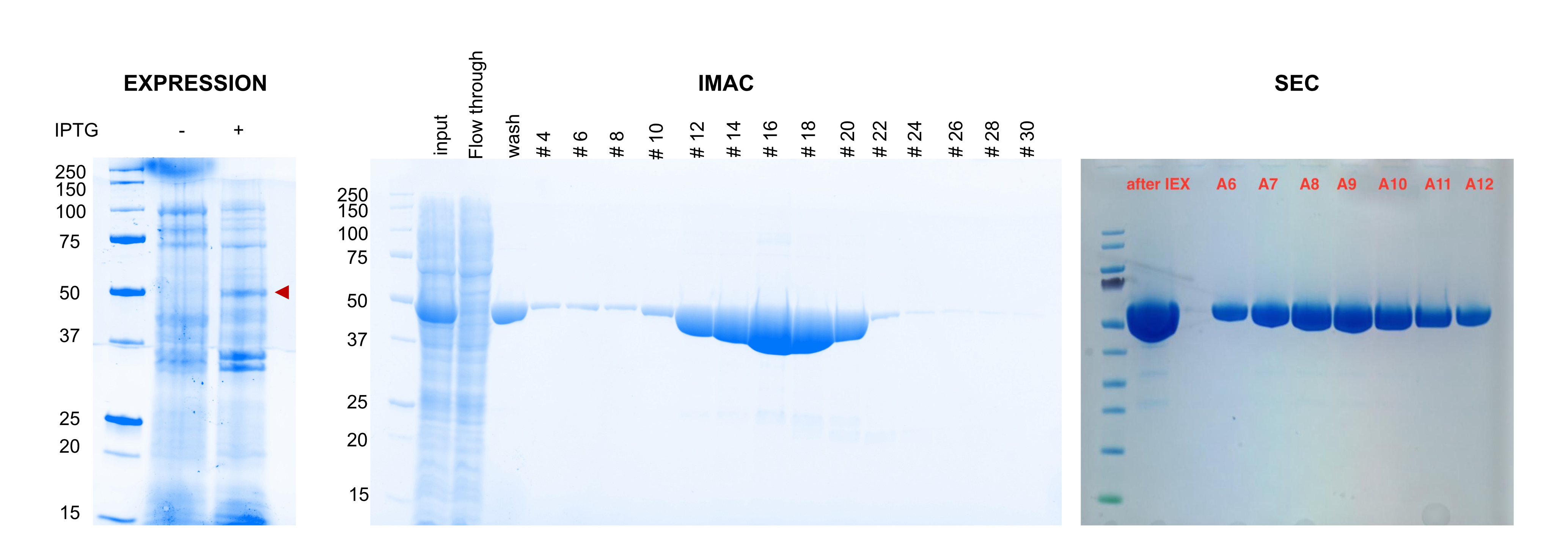


**Figure S1.** Simplified workflow of *Sb*UGT81B1 production. Left panel, SDS-PAGE analysis of equal amounts of total bacterial cell lysates from cells grown in the absence or presence of IPTG. Middle panel, SDS-PAGE analysis of samples representative from each step from IMAC purification. Right panel, SDS-PAGE analysis of *Sb*UGT85B1 before and after SEC purification. The purified protein carries a tag of six histidines at the C-terminus.


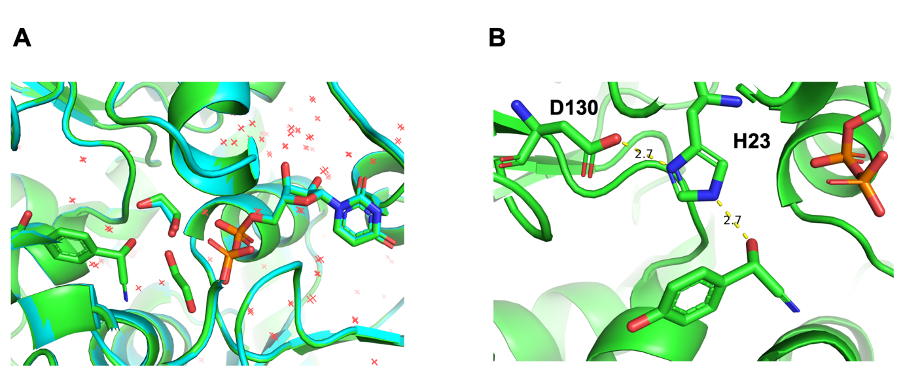


**Figure S2.** (**A**) Comparison of the two *Sb*UGT85B1 crystal structures with UDP (cyan) and (*S*)-*p*-hydroxymandelonitrile (green) bound in the active site, respectively. The two structures are very similar. (B) The His-Asp catalytic dyad in the active site of UGT85B1 close to (*S*)-*p*-hydroxymandelonitrile. Hydrogen bonds are shown with dashed yellow lines and distances are shown in angstrom (Å).


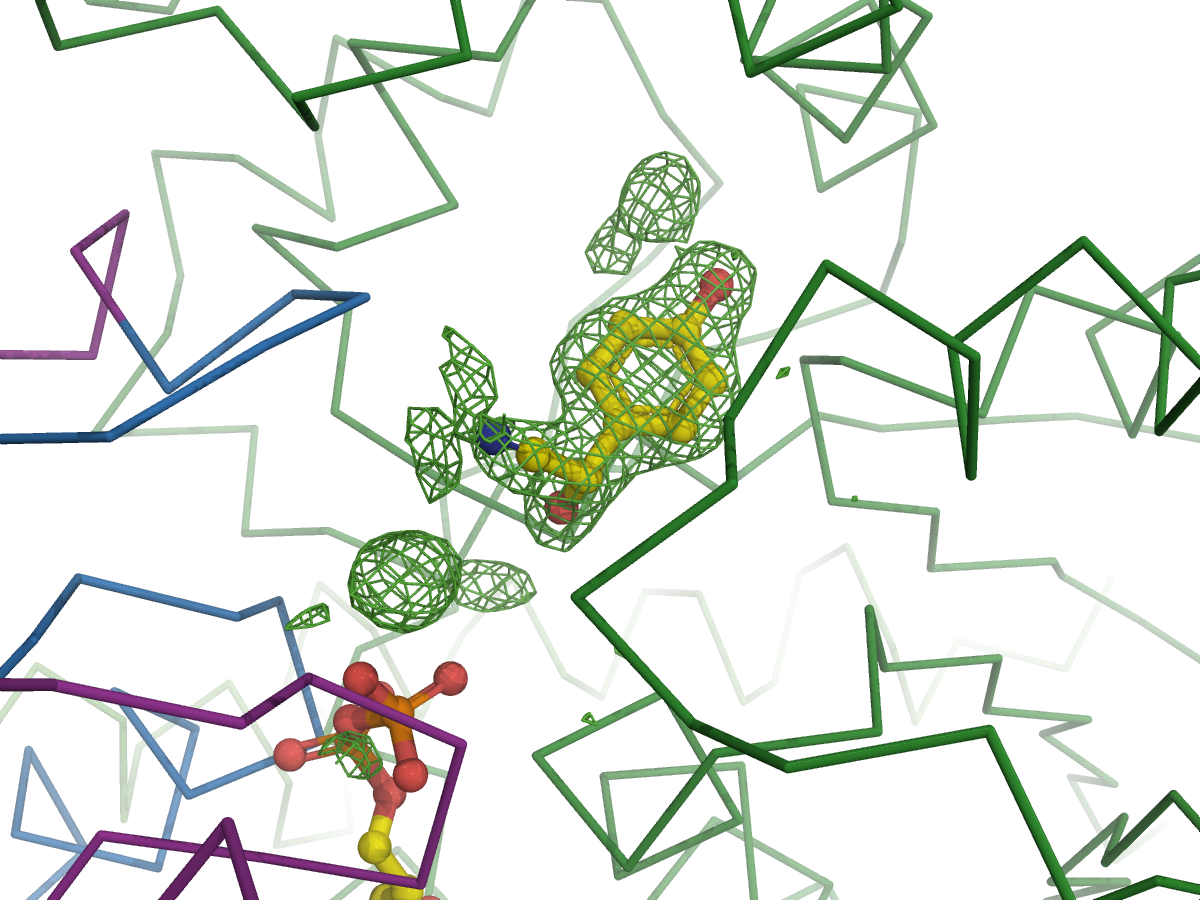


**Figure S3.** Difference electron density before modelling the (*S*)-*p*-hydroxymandelonitrile in the active site of *Sb*UGT85B1.


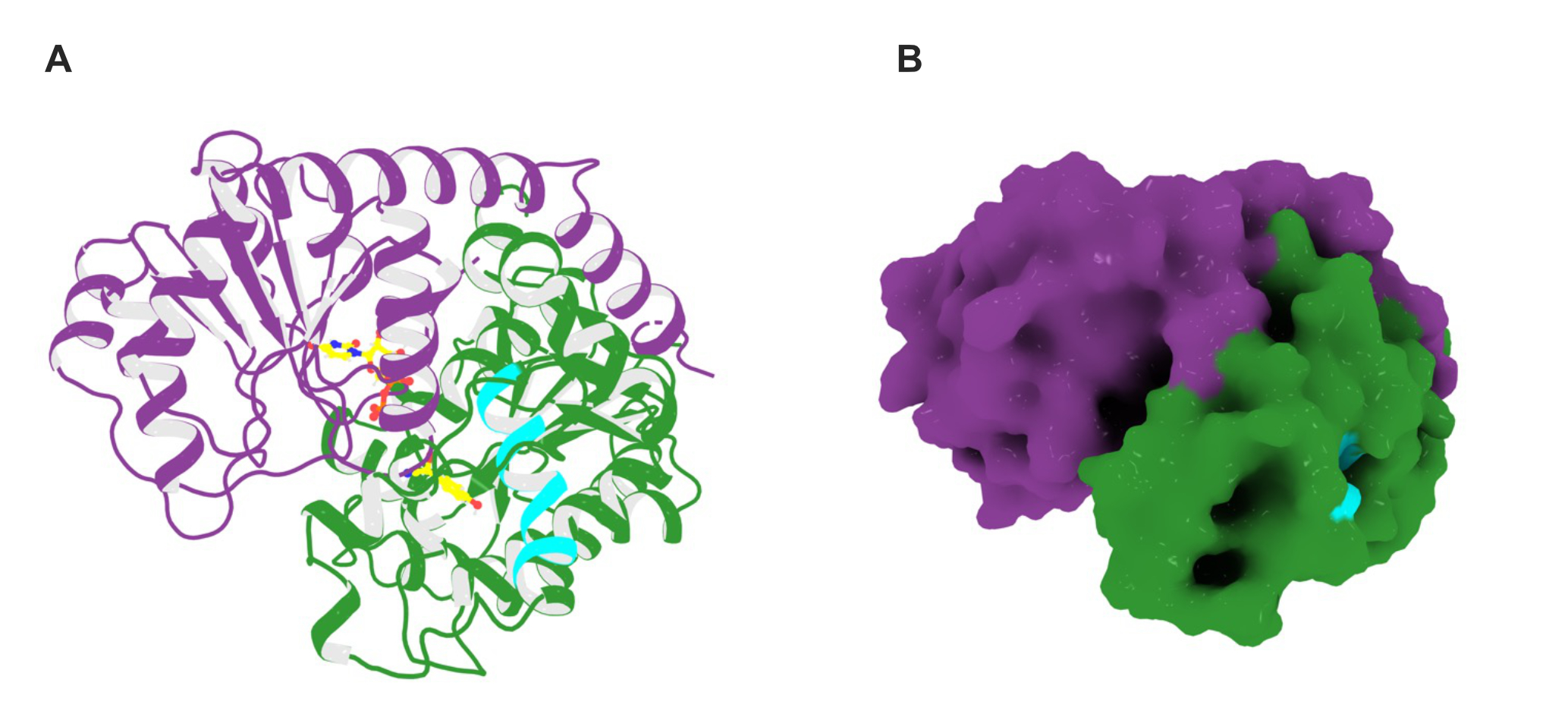


**Figure S4.** Cartoon (**A**) and surface view (**B**) (same orientation) of the *Sb*UGT85B1 structure. The hydrophobic cluster (residues 156-167) in the structure of *Sb*UGT85B1 is shown in cyan.


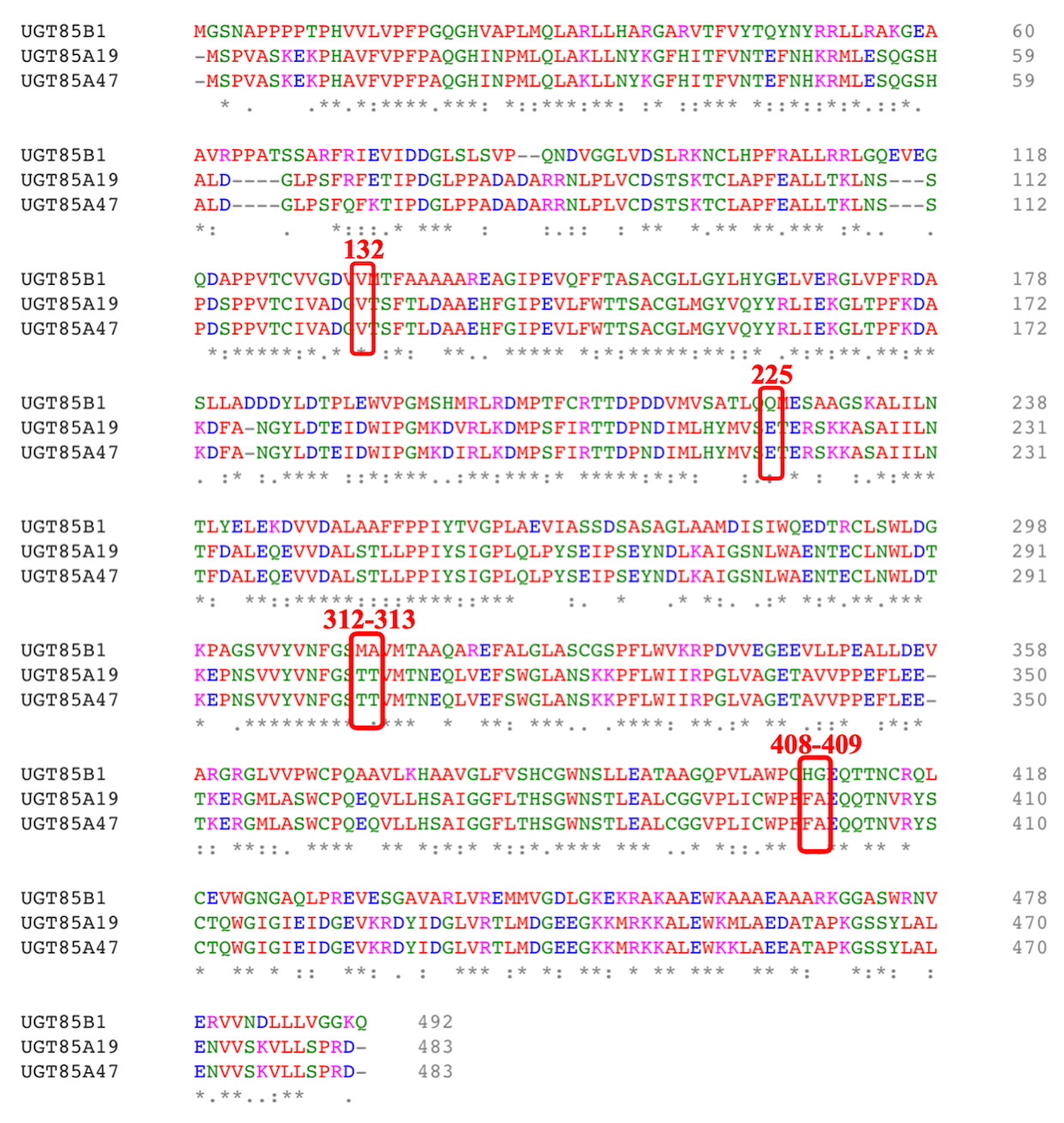


**Figure S5**. Sequence alignment on which the mutant design for altered stereospecificity was based. The red rectangle shows the residues selected for mutagenesis.

**
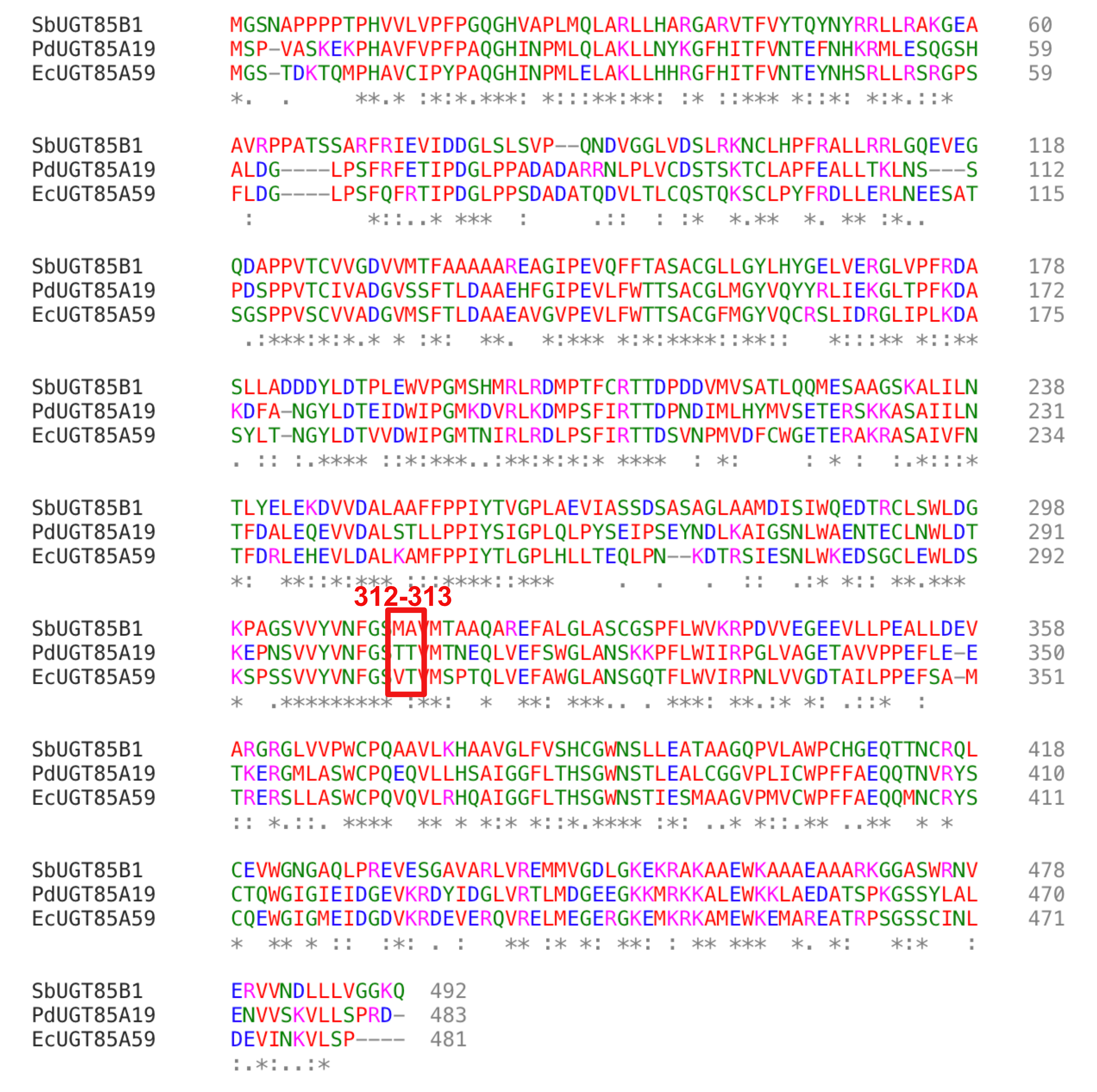
**

**Figure S6.** *Sb*UGT85B1, *Pd*UGT85A19 and *Ec*UGT85A59 sequence alignment. The red rectangle depicts the residues particularly important for the enzyme’s stereo-specificity.

***
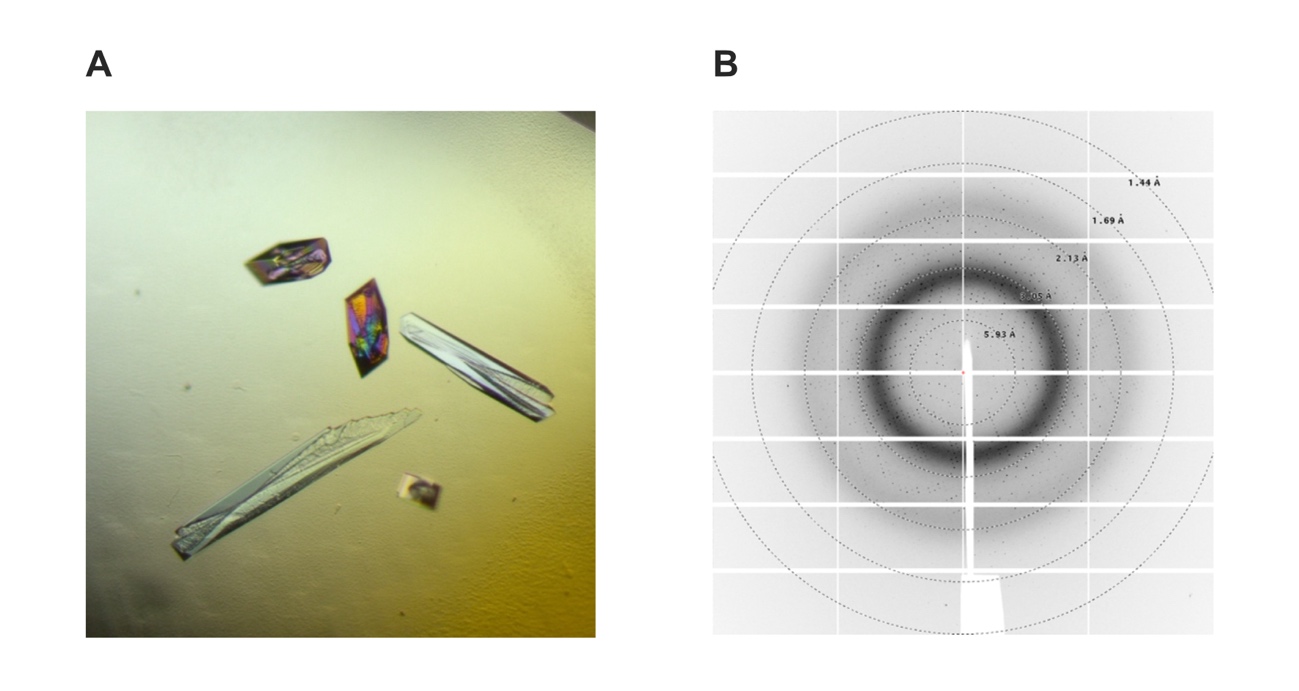
***

**Figure S7.** The *p*-hydroxymandelonitrile-soaked crystals of *Sb*UGT85B1 (**A**) and a diffraction image (**B**).


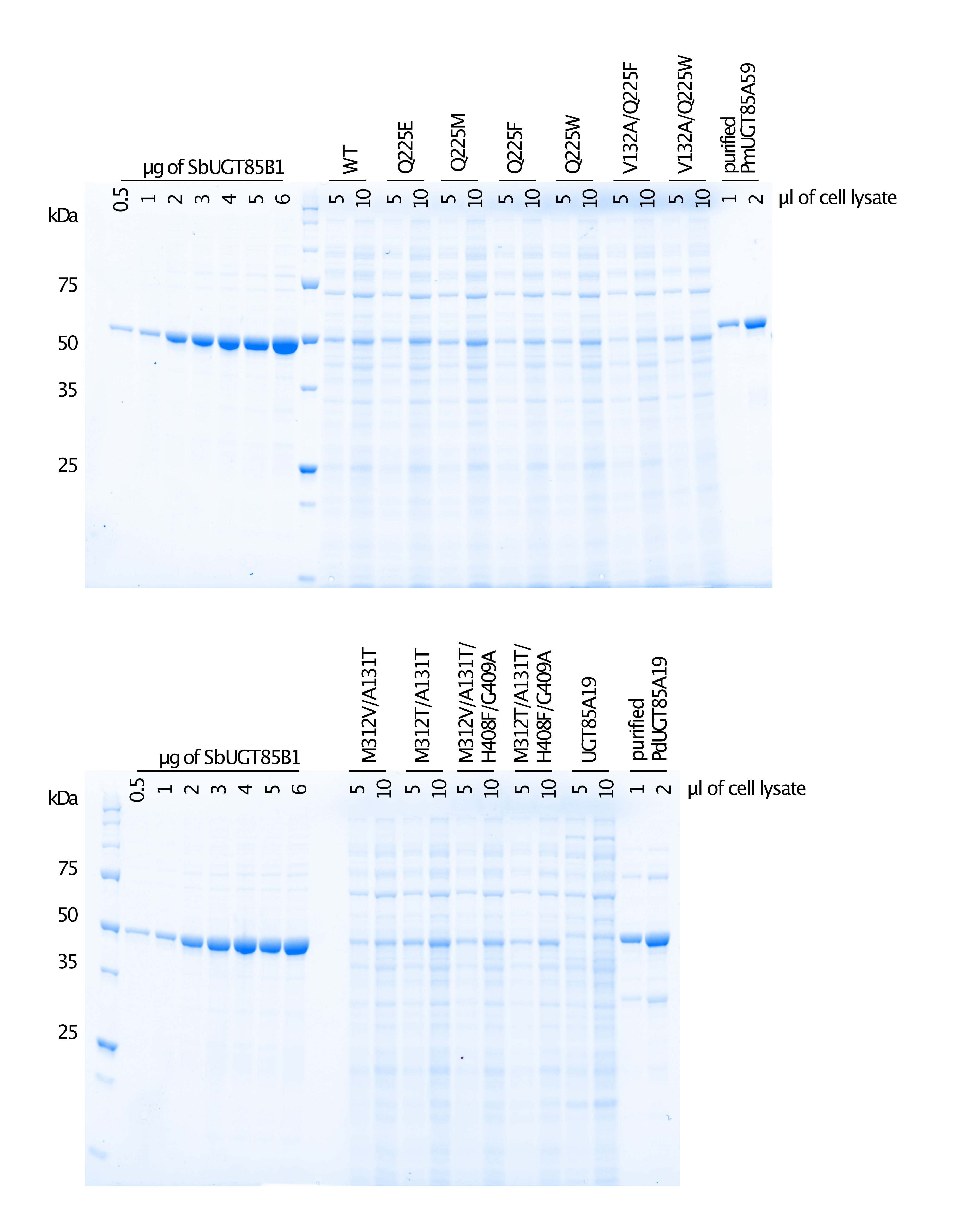


**Figure S8.** SDS-PAGE analysis of 5 and 10 μl of cell lysate from *E. coli* expressing *Sb*UGT85B1 mutants and definite amounts of pure *Sb*UGT85B1 (from 0.5 to 6 μg) used to generate a reference plot correlating protein density to protein amount.


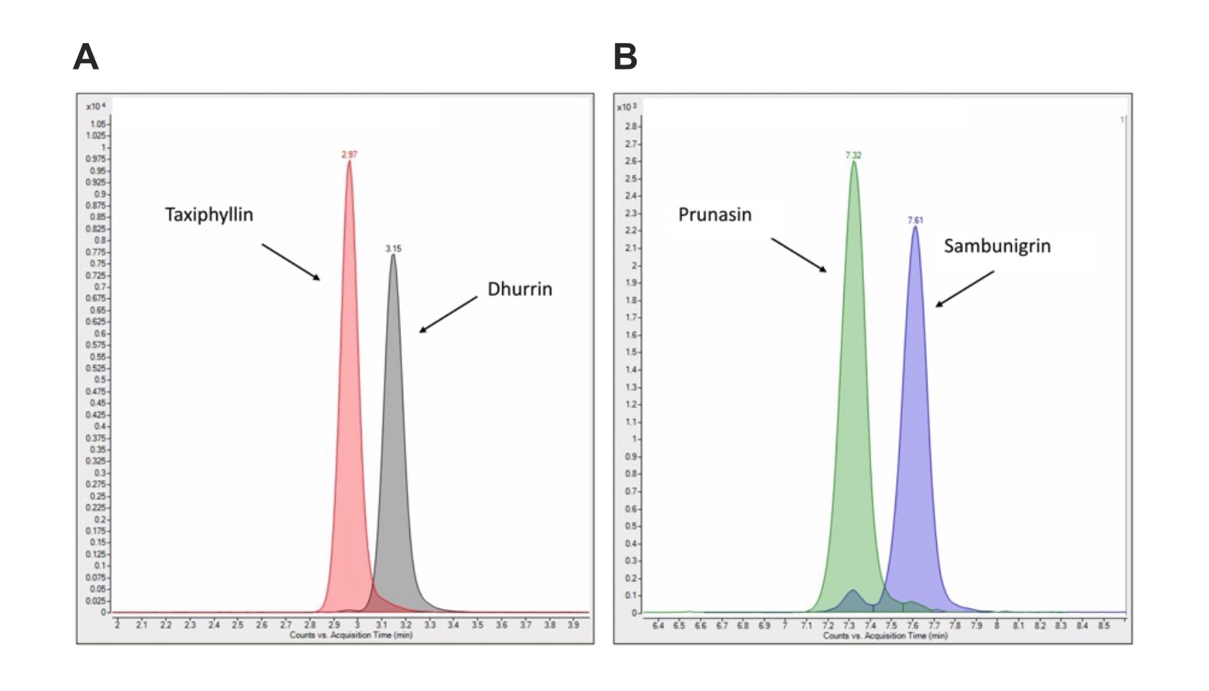


**Figure S9.** LC-MS/MS separation of the standard isomer pairs taxiphyllin - dhurrin (**A**) and prunasin – sambunigrin (**B**).

**Table S1.** Results of the two-way ANOVA, Dunnett’s multiple comparison test, performed on activity assay data reported in Figure 4

| **mandelonitrile over *p*-hydroxymandelonitrile specificity** | | | | | | |
| --- | --- | --- | --- | --- | --- | --- |
| **Dunnett's multiple comparisons test** | | **Mean Diff** | **95,00% CI of diff** | **Below threshold?** | **Summary** | **Adjusted P Value** |
| **Dhurrin** |  | |  |  |  |  |
| B1 WT vs. B1 Q225E | 2,944 | | 2,303 to 3,585 | Yes | **** | <0,0001 |
| B1 WT vs. B1 Q225M | 0,3562 | | -0,2848 to 0,9972 | No | ns | 0,5166 |
| B1 WT vs. B1 Q225F | 3,324 | | 2,683 to 3,965 | Yes | **** | <0,0001 |
| B1 WT vs. B1 Q225W | 4,991 | | 4,350 to 5,632 | Yes | **** | <0,0001 |
| B1 WT vs. B1 V132A/Q225F | 3,807 | | 3,166 to 4,448 | Yes | **** | <0,0001 |
| B1 WT vs. B1 V132A/Q225W | 5,652 | | 5,011 to 6,293 | Yes | **** | <0,0001 |
| B1 WT vs. A19 | 5,612 | | 4,971 to 6,253 | Yes | **** | <0,0001 |
| **Taxiphyllin** |  | |  |  |  |  |
| B1 WT vs. B1 Q225E | 1,699 | | 1,058 to 2,340 | Yes | **** | <0,0001 |
| B1 WT vs. B1 Q225M | 1,256 | | 0,6149 to 1,897 | Yes | **** | <0,0001 |
| B1 WT vs. B1 Q225F | 1,719 | | 1,078 to 2,360 | Yes | **** | <0,0001 |
| B1 WT vs. B1 Q225W | 1,767 | | 1,126 to 2,408 | Yes | **** | <0,0001 |
| B1 WT vs. B1 V132A/Q225F | 1,737 | | 1,096 to 2,378 | Yes | **** | <0,0001 |
| B1 WT vs. B1 V132A/Q225W | 1,774 | | 1,133 to 2,415 | Yes | **** | <0,0001 |
| B1 WT vs. A19 | -2,169 | | -2,810 to -1,528 | Yes | **** | <0,0001 |
| **Sambunigrin** |  | |  |  |  |  |
| B1 WT vs. B1 Q225E | 0,5007 | | -0,1403 to 1,142 | No | ns | 0,1861 |
| B1 WT vs. B1 Q225M | 0,1174 | | -0,5236 to 0,7583 | No | ns | 0,9951 |
| B1 WT vs. B1 Q225F | 0,2735 | | -0,3675 to 0,9145 | No | ns | 0,7653 |
| B1 WT vs. B1 Q225W | 0,4002 | | -0,2408 to 1,041 | No | ns | 0,3941 |
| B1 WT vs. B1 V132A/Q225F | 0,4716 | | -0,1694 to 1,113 | No | ns | 0,2354 |
| B1 WT vs. B1 V132A/Q225W | 0,5464 | | -0,09458 to 1,187 | No | ns | 0,1252 |
| B1 WT vs. A19 | -9,469 | | -10,11 to -8,828 | Yes | **** | <0,0001 |
| **Prunasin** |  | |  |  |  |  |
| B1 WT vs. B1 Q225E | 4,464 | | 3,823 to 5,105 | Yes | **** | <0,0001 |
| B1 WT vs. B1 Q225M | 0,1576 | | -0,4834 to 0,7986 | No | ns | 0,9792 |
| B1 WT vs. B1 Q225F | 0,4407 | | -0,2003 to 1,082 | No | ns | 0,2974 |
| B1 WT vs. B1 Q225W | 1,852 | | 1,211 to 2,493 | Yes | **** | <0,0001 |
| B1 WT vs. B1 V132A/Q225F | 2,868 | | 2,227 to 3,509 | Yes | **** | <0,0001 |
| B1 WT vs. B1 V132A/Q225W | 6,007 | | 5,366 to 6,648 | Yes | **** | <0,0001 |
| B1 WT vs. A19 | 9,792 | | 9,151 to 10,43 | Yes | **** | <0,0001 |
| **(*S*)- over (*R*)- isomer specificity** | | | | | | |
| **Dhurrin** |  | |  |  |  |  |
| B1 WT vs. B1 M312V/A313T | 0,808 | | 0,3766 to 1,239 | Yes | **** | <0,0001 |
| B1 WT vs. B1 M312T/A313T | 2,453 | | 2,022 to 2,885 | Yes | **** | <0,0001 |
| B1 WT vs. B1 M312V/A313T/H408F/G409A | 3,143 | | 2,711 to 3,574 | Yes | **** | <0,0001 |
| B1 WT vs. B1 M312T/A313T/H408F/G409A | 4,192 | | 3,760 to 4,623 | Yes | **** | <0,0001 |
| B1 WT vs. A19 | 5,612 | | 5,181 to 6,044 | Yes | **** | <0,0001 |
| **Taxiphyllin** |  | |  |  |  |  |
| B1 WT vs. B1 M312V/A313T | -0,4958 | | -0,9272 to -0,06431 | Yes | * | 0,0189 |
| B1 WT vs. B1 M312T/A313T | 0,5525 | | 0,1211 to 0,9840 | Yes | ** | 0,0074 |
| B1 WT vs. B1 M312V/A313T/H408F/G409A | -2,83 | | -3,261 to -2,398 | Yes | **** | <0,0001 |
| B1 WT vs. B1 M312T/A313T/H408F/G409A | -1,682 | | -2,114 to -1,251 | Yes | **** | <0,0001 |
| B1 WT vs. A19 | -2,169 | | -2,600 to -1,738 | Yes | **** | <0,0001 |
| **Sambunigrin** |  | |  |  |  |  |
| B1 WT vs. B1 M312V/A313T | -0,7698 | | -1,201 to -0,3384 | Yes | *** | 0,0001 |
| B1 WT vs. B1 M312T/A313T | 0,1915 | | -0,2400 to 0,6229 | No | ns | 0,6684 |
| B1 WT vs. B1 M312V/A313T/H408F/G409A | -7,262 | | -7,694 to -6,831 | Yes | **** | <0,0001 |
| B1 WT vs. B1 M312T/A313T/H408F/G409A | -5,534 | | -5,966 to -5,103 | Yes | **** | <0,0001 |
| B1 WT vs. A19 | -9,469 | | -9,900 to -9,037 | Yes | **** | <0,0001 |
| **Prunasin** |  | |  |  |  |  |
| B1 WT vs. B1 M312V/A313T | 5,865 | | 5,433 to 6,296 | Yes | **** | <0,0001 |
| B1 WT vs. B1 M312T/A313T | 8,127 | | 7,695 to 8,558 | Yes | **** | <0,0001 |
| B1 WT vs. B1 M312V/A313T/H408F/G409A | 9,094 | | 8,663 to 9,526 | Yes | **** | <0,0001 |
| B1 WT vs. B1 M312T/A313T/H408F/G409A | 9,478 | | 9,047 to 9,910 | Yes | **** | <0,0001 |
| B1 WT vs. A19 | 9,792 | | 9,361 to 10,22 | Yes | **** | <0,0001 |

ns = not significant

**Table S2.** Data collection and refinement statistics of *Sb*UGT85B1 structures

|  | **SbUGT85B1 with UDP** | **SbUGT85B1 with UDP:*p*‑hydroxymandelonitrile** |
| --- | --- | --- |
| Resolution range | 41.21 - 1.42 (1.471 - 1.42) | 45.69 - 1.5 (1.554 - 1.5) |
| Space group | P 21 21 21 | P 21 21 21 |
| Unit cell (Å) | 76.9 90.15 92.68 | 76.63 91.38 92.75 |
| Total reflections | 603276 (44070) | 581568 (57397) |
| Unique reflections | 120842 (11262) | 104569 (10304) |
| Multiplicity | 5.0 (3.9) | 5.6 (5.6) |
| Completeness (%) | 99.19 (93.40) | 99.86 (99.13) |
| Mean I/sigma(I) | 14.84 (1.01) | 13.55 (0.55) |
| Wilson B-factor | 22.02 | 29.44 |
| R-merge | 0.04583 (1.127) | 0.04737 (2.175) |
| R-meas | 0.05123 (1.304) | 0.05242 (2.4) |
| R-pim | 0.02253 (0.6415) | 0.02218 (1.004) |
| CC1/2 | 0.999 (0.426) | 0.999 (0.329) |
| CC* | 1 (0.773) | 1 (0.703) |
| Reflections used in refinement | 120818 (11244) | 104493 (10222) |
| Reflections used for R-free | 5918 (543) | 5124 (514) |
| R-work | 0.1810 (0.3430) | 0.1885 (0.4132) |
| R-free | 0.1946 (0.3429) | 0.2097 (0.4211) |
| CC(work) | 0.960 (0.694) | 0.958 (0.630) |
| CC(free) | 0.958 (0.661) | 0.957 (0.612) |
| Number of non-hydrogen atoms | 3979 | 4000 |
| macromolecules | 3599 | 3599 |
| ligands | 106 | 144 |
| solvent | 331 | 333 |
| Protein residues | 474 | 474 |
| RMS(bonds) | 0.008 | 0.010 |
| RMS(angles) | 0.97 | 1.07 |
| Ramachandran favored (%) | 97.87 | 97.87 |
| Ramachandran allowed (%) | 2.13 | 2.13 |
| Ramachandran outliers (%) | 0.00 | 0.00 |
| Rotamer outliers (%) | 0.53 | 0.80 |
| Clashscore | 2.60 | 3.95 |
| Average B-factor | 26.87 | 38.85 |
| macromolecules | 26.34 | 38.38 |
| ligands | 28.81 | 40.90 |
| solvent | 32.40 | 43.48 |

Statistics for the highest-resolution shell are shown in parentheses.

**Table S3.** List of primers used to introduce the indicated mutations in the *Sb*UGT85B1 gene. The primers were designed by using the QuikChange Primer Design tool available on Agilent website <https://www.agilent.com/store/primerDesignProgram.jsp>

| Mutation | Primers used |
| --- | --- |
| V132A | 5'-tggttggtgatgtcgcgatgacgttcgcagc-3'  5'-gctgcgaacgtcatcgcgacatcaccaacca-3' |
| Q225W | 5'-ttagcgccacgctgcagtggatggaatcagctgcggg-3'  5'-cccgcagctgattccatccactgcagcgtggcgctaa-3' |
| Q225F | 5'-ttagcgccacgctgcagttcatggaatcagctgcggg-3'  5'-cccgcagctgattccatgaactgcagcgtggcgctaa-3' |
| Q225E | 5'-gcgccacgctgcaggagatggaatcagctgc-3'  5'-gcagctgattccatctcctgcagcgtggcgc-3' |
| Q225M | 5'-ttagcgccacgctgcagatgatggaatcagctgcggg-3'  5'-cccgcagctgattccatcatctgcagcgtggcgctaa-3' |
| M312T-A313T | 5'-attttggctccacgacggtgatgacggcc-3'  5'-ggccgtcatcaccgtcgtggagccaaaat-3' |
| M312V-A313T | 5'-attttggctccgtgacggtgatgacggcc-3'  5'-ggccgtcatcaccgtcacggagccaaaat-3' |
| H408F-G409A | 5'-tctggcatggccgtgctttgctgaacagaccacgaac-3'  5'-gttcgtggtctgttcagcaaagcacggccatgccaga-3' |

**Table S4.** MRM transitions for cyanogenic glucosides quantified by LC-MS/MS

| **Analyte** | **Retention Time**  **[min]** | **Q1**  **[*m/z*]** | **Q3 [*m/z*]** | **Fragmentor**  **[V]** | **CE**  **[V]** |
| --- | --- | --- | --- | --- | --- |
| Taxiphyllin | 2.75 | 334.1 | 185.0^Qt^ | 97 | 12 |
| [M+Na]^+^ |  | 334.1 | 145.0 | 97 | 16 |
|  |  | 334.1 | 307.0 | 97 | 12 |
| Dhurrin | 3,16 | 334.1 | 185.0^Qt^ | 97 | 12 |
| [M+Na]^+^ |  | 334.1 | 145.0 | 97 | 16 |
|  |  | 334.1 | 307.0 | 97 | 12 |
| Prunasin | 7.33 | 318.1 | 185.0^Qt^ | 83 | 12 |
| [M+H]^+^ |  | 447.0 | 129.0 | 83 | 12 |
|  |  | 447.0 | 97.0 | 83 | 24 |
| Sambunigrin | 7.62 | 318.1 | 185.0^Qt^ | 83 | 12 |
| [[M+H]^+^ |  | 447.0 | 129.0 | 83 | 12 |
|  |  | 447.0 | 97.0 | 83 | 24 |

Qt = quantifier ion, additional transitions were used for identification. Q = quadrupole. CE = collision energy.

**Supporting Materials and Methods**

**Chemical synthesis of *p*-hydroxymandelonitrile [2-hydroxy-2-(4-hydroxyphenyl)acetonitrile]**

A mixture of *p*-hydroxybenaldehyde (0.92, 7.5 mmol), solid LiClO_4_ (1.62 g, 15.2 mmol) and trimethylsilyl cyanide (TMS-CN) (2.4 mL, 20.0 mmol) was stirred at room temperature (r.t.) for 2 h. Then CH_2_Cl_2_ was added to the reaction mixture and the lithium perchlorate was filtered off. The organic layer was washed with water, and then dried over anhydrous MgSO_4_. The solvent was removed by a rotary evaporator to obtain almost pure crude product of 2,4’-di-*O*-trimethylsilyl cyanohydrin derivative. Tetrahydrofuran (THF: 6.0 mL) was added to the crude 2,4’-di-*O*-trimethylsilyl cyanohydrin, followed by the addition of hydrochloric acid (4.0 mL, 3M). The mixture was heated at 65°C (oil bath temperature) for 15 min. The reaction mixture was cooled to r.t., then diluted with ethyl acetate (50 ml) and water (25.0 mL) was added. The organic phase was washed sequentially with H_2_O (3 x 25 ml). NaHCO_3_ (25 ml), H_2_O, brine (25 ml) and dried. The solvent was removed by evaporation on a rotary evaporator and the residue was recrystallized from CH_2_Cl_2_ to give chromatographically pure an epimeric mixture of *p*-hydroxymandelonitrile as white powder (1.1 g, 98%; *E*/*Z* = 4:8).
